# Bacterial toxin sensing induces transient inflammasome-dependent innate immune memory that improves survival during sepsis

**DOI:** 10.1038/s41467-026-78499-5

**Published:** 2026-10-08

**Authors:** Cédric Torre, Valentine Marion, Zakaryia Caillot, Ludovic Batistic, Margot Saissi, Océane Dufies, Els Verhoeyen, Grégory Michel, Patrick Munro, Céline Loubatier, Anne Doye, Paul Chaintreuil, Alexandre Gallerand, Stoyan Ivanov, Dorota Czerucka, Orane Visvikis, Bénédicte F. Py, Jessica Quintin, Laurent Boyer

**Affiliations:** 1 Université Côte d’Azur, INSERM, Nice, France; 2 Harvard Medical School and Boston Children’s Hospital, Division of Immunology, Boston, MA USA; 3 CIRI, Centre International de Recherche en Infectiologie, Univ Lyon, Inserm, U1111, Université Claude Bernard Lyon 1, CNRS, UMR5308, ENS de Lyon, Lyon, France; 4 Université Côte d’Azur, CNRS, Nice, France; 5 Medical Biology Department, Centre Scientifique de Monaco, CSM, Monaco, Monaco; 6 LIA ROPSE, Laboratoire International Associé Université Côte d’Azur – Centre Scientifique de, Monaco, Monaco; 7 Institut Pasteur, Université Paris Cité, Inserm U1359, Immunology of Fungal Infections –, Paris, France; 8 Université Côte d’Azur, CHU Nice, Laboratoire de Virologie, Nice, France

**Keywords:** Pathogens, Infectious diseases, NOD-like receptors

## Abstract

Adaptive immune memory generated by bacterial toxin vaccination is well established, but whether bacterial toxins can also induce innate immune memory, or trained immunity (TI), remains unclear. Here, we show that bacterial toxins elicit TI through NLRP3 inflammasome activation both in vitro and in vivo in mice, and that this response is conserved in primary human macrophages. Stimulation with the CNF1 toxin induced an epigenetic remodeling, defining a TI-associated transcriptional signature. A targeted RNAi screen identified Bcl2a1 as a required mediator of TI, and epistasis analyses demonstrated that IL-1 signaling is essential for TI induction in macrophages. In our mouse sepsis model, toxin-induced TI enhanced host resistance and improved survival. These findings establish toxin-driven inflammasome activation as trigger of transient TI and demonstrate that this response enhance resistance during experimental bacterial sepsis.

## Abstract (web summary)

This study shows that the bacterial toxin CNF1 can train innate immune cells to respond more effectively to subsequent infections, revealing a form of immune memory that improves survival during sepsis in mice.

## Introduction

Both components of the immune system, innate and adaptive, possess the ability to remember previous encounters with pathogens. In both systems, this memory depends on the initial detection of these microbes by the innate immune system. The innate immune system of the host has adapted diverse strategies to identify pathogens, mount immediate responses, and establish a form of immune memory to these encounters. A hallmark of many pathogenic bacteria is their production of toxins or virulence factors, which play a critical role in their ability to cause disease. Consequently, a key aspect of protection against pathogenic bacteria is the development of immune memory specifically targeting these toxins. Adaptive immune memory against bacterial toxins, mediated by B and T lymphocytes, has been extensively studied^1,2^. This research has driven significant advancements in public health, including the development of effective and safe anti-toxin vaccines, such as those for tetanus and diphtheria^3^.

In contrast to adaptive immune memory, the molecular mechanisms underlying innate immune memory in response to bacterial toxins remain poorly understood^4^. This gap in knowledge highlights the need for further research to elucidate how innate immunity contributes to long-term defense against these toxins.

Toxins encoded by microbes are specific weapons by which pathogens hijack host cell signaling and circumvent the host defense to survive and multiply. Consequently, hosts have evolved sensors, which allow the detection and evaluation of the pathogenic potential of a microbe. Among these sensors, inflammasomes are cytosolic detectors of virulence factors^5^.

Inflammasomes are multiprotein platforms that allow the maturation of the proinflammatory cytokines IL-1β and IL-18^6^. They have been shown to detect PAMPs, DAMPS and more recently the activity of virulence factors, a mechanism termed effector-triggered immunity^5,7^. Virulence factors and toxins targeting Rho GTPases have been proven to fall in this last category. Pyrin and NLRP3 (NACHT LRR and pyrin domain containing 3) inflammasomes detect respectively the inhibition of RhoA and the activation of Rac2, two proteins of the Rho GTPases family targeted by microbial virulence factors^7,8^. This immune response triggered by microbial virulence factors is called effector-triggered immunity (ETI), and is added to microbial pattern-triggered immunity (PTI) in order to finely assess the quantity and pathogenicity of microbes^9,10^. While the detection of the enzymatic activities of virulence factors by inflammasomes has been established, the long-term consequences of this detection for the innate immune system have not yet been addressed. Specifically, it remains unknown how an initial microbial challenge sensed by inflammasomes affects the cells’ ability to respond to future insults.

The dogma that only adaptive immunity has immune memory in mammals was challenged more than a decade ago with the characterization of trained immunity (TI)^11–13^. TI is a form of innate immune memory mediated by innate immune cells and is characterized by a potentiated immune response after a first encounter with a TI-inducing stimulus. The underlying mechanisms of TI involve metabolic and epigenetic reprogramming at the cellular level^12–14^, and recent work has highlighted the importance of the IL-1 signaling pathway in its establishment at the organismal level^4,15^. An inflammatory diet has been shown to induce TI via the NLRP3 inflammasome in a *Ldlr*^−/−^ mouse model susceptible to the development of atherosclerosis^16^. However, the role of NLRP3 inflammasome–driven TI in the context of host-pathogen interaction still needs to be characterized.

Here, we identified a trained immune protection against sepsis triggered by the *Escherichia coli* toxin CNF1, a Rho GTPase-targeting toxin, mediated through NLRP3 inflammasome activation, and we identified the BCL2A1 protein as a crucial molecular player in the establishment of this effector-triggered trained immunity.

## Results

### CNF1 toxin induces trained immunity

**Fig. 1:**
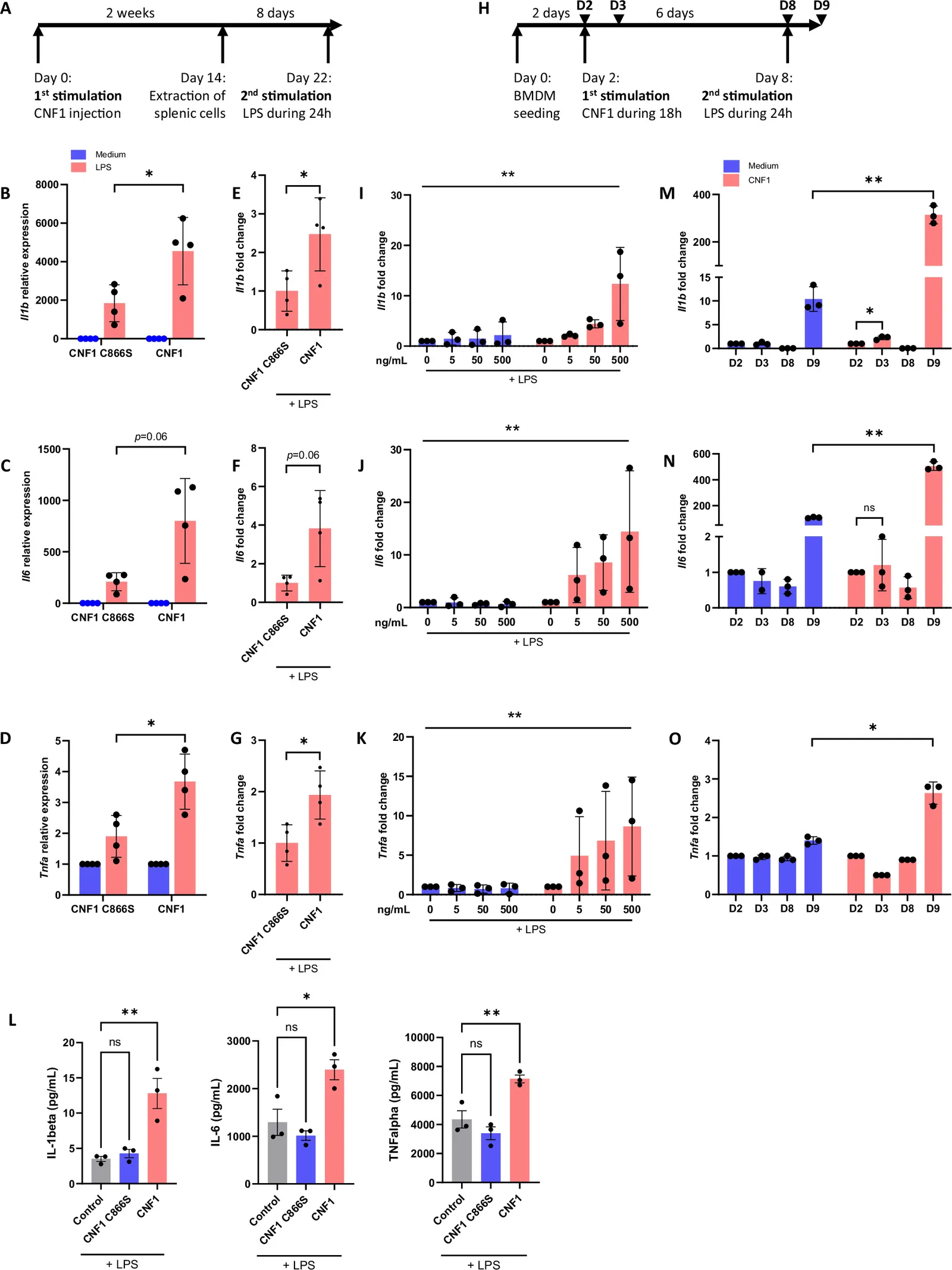
CNF1 toxin induces trained immunity in vivo and in vitro. **A** Experimental setting for the induction of trained immunity (TI) in C57BL/6 J mice with the CNF1 toxin. **B**–**G** WT C57BL/6 J mice were trained with intravenous injection of CNF1 (*n* = 4) or its catalytic inactive mutant (CNF1^C866S^) (250 ng/kg) (*n* = 4) before splenic macrophages were isolated, cultured then treated with LPS for 24 h (10 ng/mL). Gene expression were analyzed using qPCR for *Il1b* ((**B**) *p* = 0.0454, (**E**) *p* = 0.0452), *Il6* (**C**, **F**) and *Tnfa* ((**D**) *p* = 0.0213, (**G**) *p* = 0.0210), expressed in relative expression (**B**–**D**) and fold change (**E**–**G**). Experiments were repeated three times. **H** Experimental setting for the induction of TI in bone marrow-derived macrophages (BMDM) isolated from WT C57Bl/6 mice. **I**–**K** BMDM isolated from WT C57BL/6 J mice were trained with CNF1 or its catalytic inactive mutant at different concentrations (5, 50, 500 ng/mL) for 18 h before treatment with LPS for 24 h (10 ng/mL). *N* = 3 for the different conditions. Gene expression were analyzed using qPCR for *Il1b* ((**I**) *p* = 0.0018), *Il6* ((**J**) *p* = 0.0038) and *Tnfa* ((**K**) *p* = 0.007). Experiments were repeated three times. **L** Mice BMDMs were trained with CNF1 (5 ng/mL) (*n* = 3) or the mutant CNF1 C866S (5 ng/mL) (*n* = 3) on day two. Control BMDMs (*n* = 3) were not trained. On day 8, all BMDMs were treated with LPS (10 ng/mL) for 6 h and secreted levels of IL-1β (** corresponds to *p* = 0.004 and ns to *p* = 0.8835), IL-6 (* corresponds to *p* = 0.0167 and ns to *p* = 0.5671) and TNFα (** corresponds to *p* = 0.0089 and ns to *p* = 0.3053) were assessed in the medium. The experiment was repeated three times. **M**–**O** BMDM isolated from WT C57BL/6 J mice were trained (*n* = 3) or not (*n* = 3) with CNF1 (50 ng/mL) for 18 h before treatment with LPS for 24 h (10 ng/mL). Gene expression were analyzed using qPCR for *Il1b* (**L**), *Il6* (M) and *Tnfa* (**N**) at different time points (before (D2) and after (D3) the first stimulation (CNF1), and before (D8) and after (D9) the second stimulation (LPS). **M** ** corresponds to *p* = 0.005 and * corresponds to *p* = 0.03. **N** ** corresponds to *p* = 0.0019 and ns corresponds to *p* = 0.6784. (**O**) * corresponds to *p* = 0.109. (Data are means ± SEM. T-tests have been used for pairwise comparisons and ANOVA for group comparisons, with results considered statistically significant if *p* ≤ 0.05 (*p* ≤ 0.05: * - ≤ 0.01: ** - ≤ 0.001: ***). Two-sided tests were used. Experiments were repeated three times.

We set up an assay to monitor the long-term effect of the CNF1 toxin-triggered NLRP3 inflammasome activation in vivo. To this aim, mice were injected intravenously with the recombinant CNF1 toxin or the catalytic inactive mutant C866S form (Fig. 1A). Two weeks after the injection, splenic macrophages were isolated and cultured for 8 additional days. We focused on splenic macrophages because they are key sentinels for the detection and clearance of blood-borne pathogens and play a critical role in orchestrating immune responses during systemic infections, making them a relevant population to investigate in this context^17,18^. Macrophages were then stimulated with the same amount of lipopolysaccharide (LPS) for 24 h before analysis of IL-1β, IL-6 and TNFα cytokine expression. We measured a significant increase for both IL-1β and TNFα cytokine expression as well as a trend toward increased IL-6 expression in macrophages isolated from mice previously injected (more than three weeks before) with the CNF1 toxin compared to the catalytic inactive mutant (Fig. 1B–G). We used the catalytically inactive CNF1 mutant as a control, as it exhibited purity, solubility, and stability under various storage conditions comparable to those of wild-type CNF1 (Suppl. Fig. 1A–C). We also confirmed in our cellular system that the catalytically inactive CNF1 mutant, unlike wild-type CNF1, failed to induce Rac activation both in HEK293T cells and in bone marrow-derived macrophages (BMDM) isolated from naïve mice (Suppl. Fig. 1C, D). Accordingly, we measured the secretion levels of IL-1β proinflammatory cytokine of treated BMDM and observed similar fold-changes with either CNF1 C866S-injected mice or PBS-injected mice when used as a normalizer in contrast to the induction observed with wild type CNF1 (Suppl. Fig. 1E). We pointed out similarities with the previously observed trained immunity (TI) phenotype induced by BCG or β-glucans^12,13^. To further confirm that our phenotype was related to TI we investigated whether the CNF1-triggered TI was cell autonomous. To this aim, we used BMDM extracted from naive mice and proceeded based on a previously described protocol^16^. BMDM were differentiated into macrophages using CSF-1. At day 2, cells were treated with different amounts of CNF1 toxin or the catalytic inactive mutant for 18 h. Macrophages were then stimulated 6 days later using LPS for 24 h (Fig. 1H). Though macrophages were all treated with the same amount of LPS, we measured a dose-dependent increase of IL-1β, IL-6 and TNFα cytokine expression in macrophages treated with different concentrations of the CNF1 toxin one week before (Fig. 1I–K). Consistent with these results, we also measured a higher amount of IL-1β, IL-6 and TNFα cytokines secretion in the supernatant of macrophages treated with CNF1 toxin compared to the mutant CNF1 C8666S or untreated control cells (Fig. 1L-N). To confirm the TI phenotype, we took advantage of this cellular model of TI to measure the expression of IL-1β, IL-6 and TNFα at different time points, before and after stimulation with the CNF1 toxin and before and after stimulation with LPS, on days 2, 3, 8 and 9, respectively (Fig. 1H). We measured a significant increase in IL-1β and a trend of increase in IL-6 expression induced by stimulation with CNF1 toxin, a decrease in their expression before stimulation with LPS, and then an exacerbated expression of IL-1β, IL-6 and TNFα after stimulation with LPS (Fig. 1O–Q). This expression pattern of proinflammatory cytokines corresponds to the current definition of TI and thus confirms that the phenotype induced by the CNF1 toxin is a bona fide trained immunity^19^.

### CNF1-induced trained immunity relies on the NLRP3 inflammasome and IL-1β signaling

**Fig. 2:**
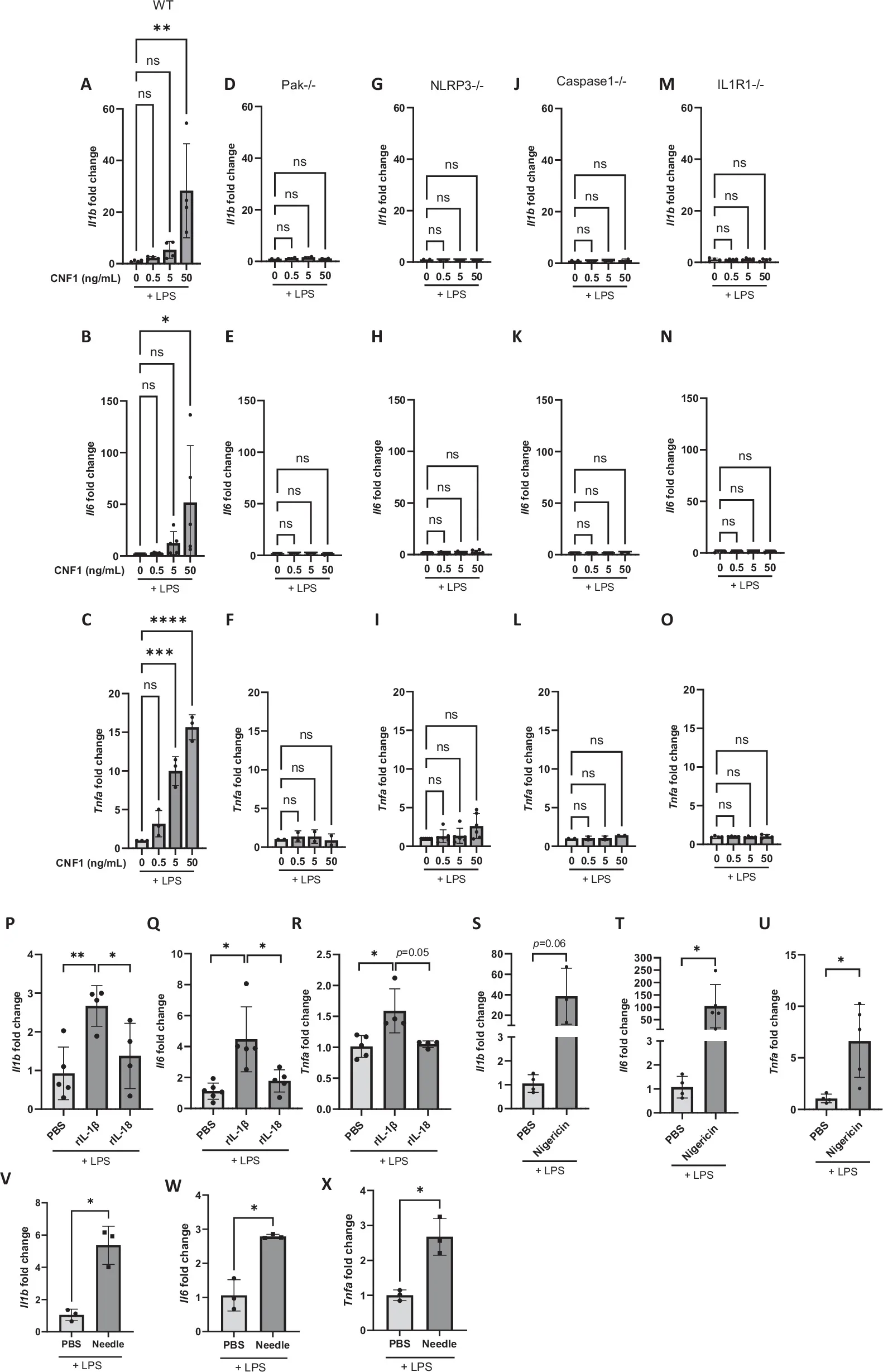
CNF1-induced TI relies on the NLRP3 inflammasome pathway. **A**–**O** BMDM isolated from WT, Pak1-, NLRP3-, Casp1/11- and IL1R1-knockout C57BL/6 J mice were trained with CNF1 at different concentrations (0.5, 5, 50 ng/mL) for 18 h before treatment with LPS for 24 h (10 ng/mL). Genes expression were analyzed using qPCR for *Il1b*, *Il6* and *Tnfa*. (**A**) ** corresponds to *p* = 0.0041, (**B**) * corresponds to *p* = 0.0373, (**C**) **** corresponds to *p* < 0.0001. **P**–**R** BMDM isolated from WT C57BL/6 J mice were trained with recombinant mouse IL-1β (10 pg/mL) or IL-18 (10 pg/mL) for 18 h before treatment with LPS for 24 h (10 ng/mL). Genes expression were analyzed using qPCR for *Il1b* (**P**), *Il6* (**Q**) and *Tnfa* (**R**). **P** ** corresponds to *p* = 0.0034 and * corresponds to *p* = 0.0479, (**Q**) * corresponds to *p* = 0.0216 and *p* = 0.429 from left to right on the graph respectively, (**R**) * corresponds to *p* = 0.039. **S–U** BMDM isolated from WT C57BL/6 J mice were trained with Nigericin (100 nM) for 18 h before treatment with LPS for 24 h (10 ng/mL). (**T**) *p* = 0.0159, (**U**) *p* = 0.0235. Genes expression were analyzed using qPCR for *Il1b* (**S**), *Il6* (**T**) and *Tnfa* (**U**). **V**–**X** BMDM isolated from WT C57BL/6 J mice were trained with Needle (2 ng/mL) for 18 h before treatment with LPS for 24 h (10 ng/mL). **V** *p* = 0.0175, (**W**) *p* = 0.0209, (**X**) *p* = 0.0246. Data are means ± SEM. T-tests have been used for pairwise comparisons, with results considered statistically significant if *p* ≤ 0.05 (*p* ≤ 0.05: * - ≤ 0.01: **). Two-sided tests were used. Experiments were repeated at least three times.

We previously showed the CNF1 sensing by the NLRP3 inflammasome was resulting from a signaling cascade involving the kinase PAK^7^. We thus used primary macrophages derived from the bone marrow of wild type mice (WT) or knock-out mice (KO) for either *Pak1*, *Nlrp3* or *Casp1/11* in our model of CNF1-triggered TI. In contrast to the dose dependent CNF1-triggered trained phenotype observed in control wild-type macrophages (Fig. 2A–C), the IL-1β, IL-6 and TNFα cytokines expression was blunted in *Pak1*^−/−^, *Nlrp3*^−/−^ or *Casp1/11*^−/−^ macrophages (Fig. 2D–O). Since the inflammasome pathway activation controls IL-1β secretion, we next asked whether the IL-1 Receptor 1 (IL-1R1) was important for the trained phenotype. We thus used macrophage KO for *Il1r1*. The CNF1-triggered trained phenotype was not observed in the macrophages *Il1r1*^−/−^, with an impaired expression of IL-1β, IL-6 and TNFα cytokines. This observation led us to investigate whether the trained phenotype we observed in our model could be initiated by IL-1 receptor stimulation using recombinant IL-1β. We observed a trained phenotype with an increased IL-1β, IL-6 and TNFα cytokine expression when we stimulated macrophages with recombinant IL-1β one week before LPS stimulation. Importantly, the trained phenotype was not observed when we used recombinant IL-18 (Fig. 2P–R). These data let us think that other NLRP3 inflammasome triggers leading to IL-1β secretion should induce a trained phenotype. To prove this point, we used Nigericin – a bona fide NLRP3 inflammasome trigger – using the same experimental setting. We observed a trained phenotype with an increased IL-1β, IL-6 and TNFα cytokine expression when macrophages were stimulated by Nigericin (Fig. 2S–U). We then investigated if innate training is a general consequence of inflammasome activation and IL-1β secretion and if it can be induced by other inflammasomes activated by bacterial signals. We used Needle as first stimulation, a protein of bacterial type III secretion systems (T3SS), known to trigger NLRC4 inflammasome. It induced an increased expression of IL-1β, IL-6 and TNFα (Fig. 2V–X). Therefore, Needle is also able to induce trained immunity indicating that training might be a general consequence of inflammasome activation and subsequent IL-1β secretion.

### CNF1-induced epigenetic and transcriptomic reprogramming

**Fig. 3:**
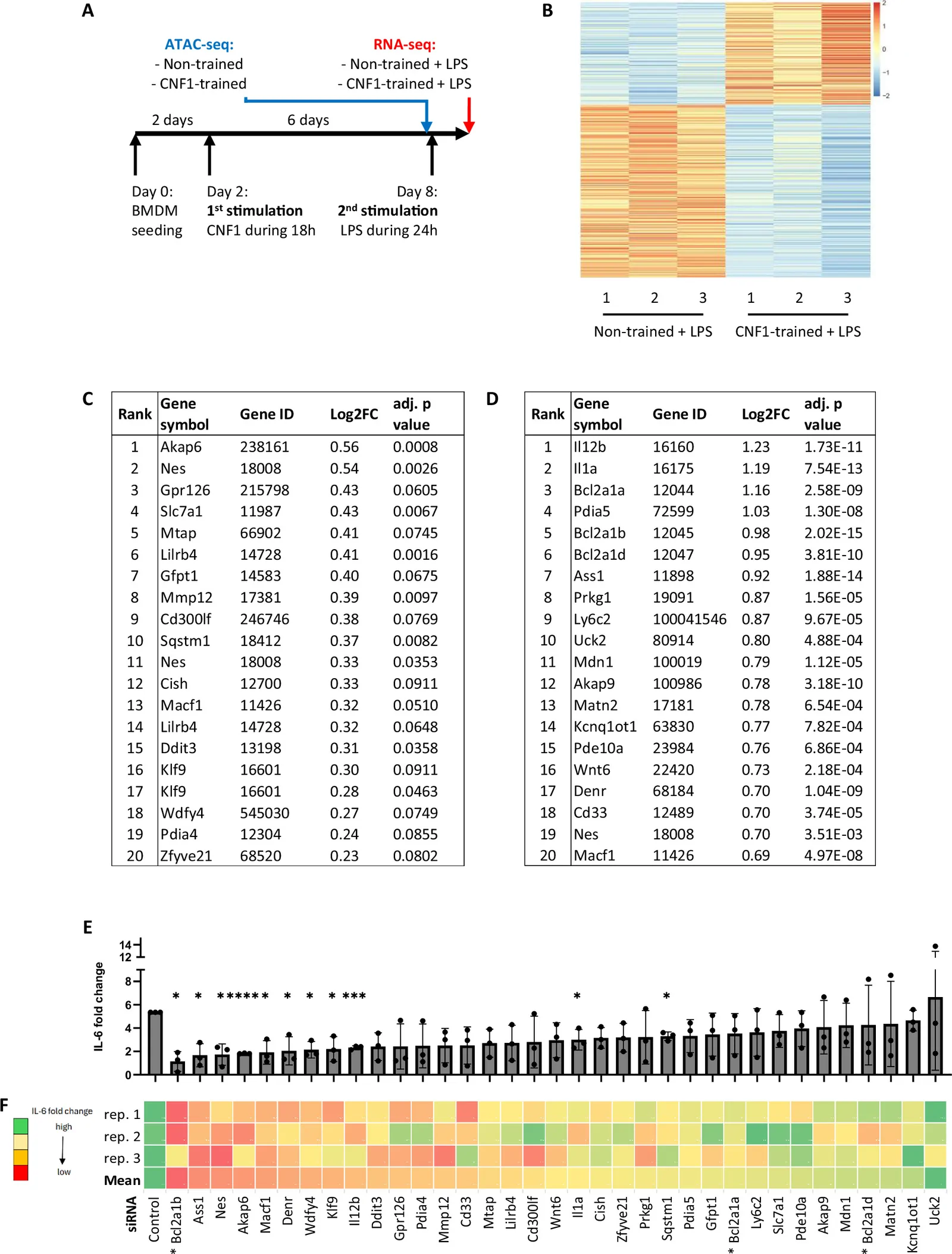
CNF1 toxin-induced TI relies on *Bcl2a1* expression. **A** Experimental setting for ATAC-seq and RNA-seq on BMDM isolated from WT C57BL/6 J mice. **B** BMDM isolated from WT C57BL/6 J mice were trained with CNF1 (50 ng/mL) for 18 h before treatment with LPS for 24 h (10 ng/mL). Gene expression were analyzed using RNA-seq. On the heatmap, red colors represent up-regulated genes and blue colors represent down-regulated genes. Scale is in Log2FC. **C**, **D** Tables of best ATAC-seq (**C**) and RNA-seq (**D**) hits. A Wald statistic test has been performed to determine *p*-value by DESeq2. Adjusted *p*-values for the differential analysis have been obtained using the Benjamini-Hochberg FDR correction method. For the ATAC-seq, a *p*-value ≤ 0.1 was considered statistically significant, and for the RNA-seq, a *p*-value ≤ 0.05 was considered statistically significant. Experiments were repeated three times. **E**, **F** BMDM isolated from WT C57BL/6 J mice were trained with CNF1 (50 ng/mL) for 18 h, transfected with the indicated siRNA for 72 h before treatment with LPS for 24 h (10 ng/mL). Supernatants were analyzed using enzyme-linked immunosorbent assay (ELISA) for IL-6. IL-6 fold change was calculated relative to non-trained - LPS stimulated BMDM. Data are means ± SEM in (**A**) and a heatmap with green color corresponding to high values and red color to low values in (**B**). Highlighted by the asterisks in B are the three Bcl2a1 family genes.

TI involves epigenetic reprogramming at the cellular level. To directly address this point, we set up a system to identify the epigenetic signature of the CNF1-triggered trained immune response. Wild-type primary macrophages were treated as previously described to induce CNF1-triggered TI. An ATAC-seq was performed 6 days after the CNF1 toxin stimulation and before the LPS stimulation to identify the CNF1-epigenetic signature. A RNA-seq analysis was performed 24 h after the LPS stimulation allowing to monitor the transcriptional signature of the cells previously exposed to the CNF1 toxin (CNF1-trained) (Fig. 3A). Confirming our hypothesis, we observed a specific pool of transcripts upregulated in the CNF1-trained cells compared to control (Fig. 3B). We also observe changes in chromatin accessibility prior to LPS treatment (Fig. 3C). This combined analysis allowed us to identify the top20 genes found to be the most accessible and significantly upregulated from the ATAC-Seq (Fig. 3C) as well as the top20 most expressed genes from the RNA-Seq experiments (Fig. 3D). We hypothesized that these more accessible and upregulated genes are important for the CNF1-triggered trained immune phenotype.

To investigate whether these top ATAC-seq and RNA-seq genes (Fig. 3C, D) were important for the CNF1-triggered trained response, we performed a functional screen by knocking down all these hits using siRNA (Fig. 3E). We found that siRNA targeting *Bcl2a1b* was inducing the highest impairment of the CNF1-triggered TI monitored here by analyzing the IL-6 cytokine secretion (Fig. 3F). Reinforcing our interest in *Bcl2a1*, all functional Bcl2a1 family members: *Bcl2a1a*, *Bcl2a1b* and *Bcl2a1d* (*Bcl2a1c* is a pseudogene) showed reduced IL-6 secretion upon knockdown, although to different extents. Importantly, RNA-seq analysis revealed that these same Bcl2a1 family members were transcriptionally induced following CNF1-mediated training, consistent with coordinated regulation of the Bcl2a1 locus. Previous studies have shown that these Bcl2a1 family members encode functional pro-survival proteins with overlapping activities^20^, supporting the biological relevance of this pathway in TI. Together, these findings led us to prioritize Bcl2a1 as a key regulator of CNF1-induced TI.

### Bcl2a1 is a key regulator of CNF1-induced trained immunity

**Fig. 4:**
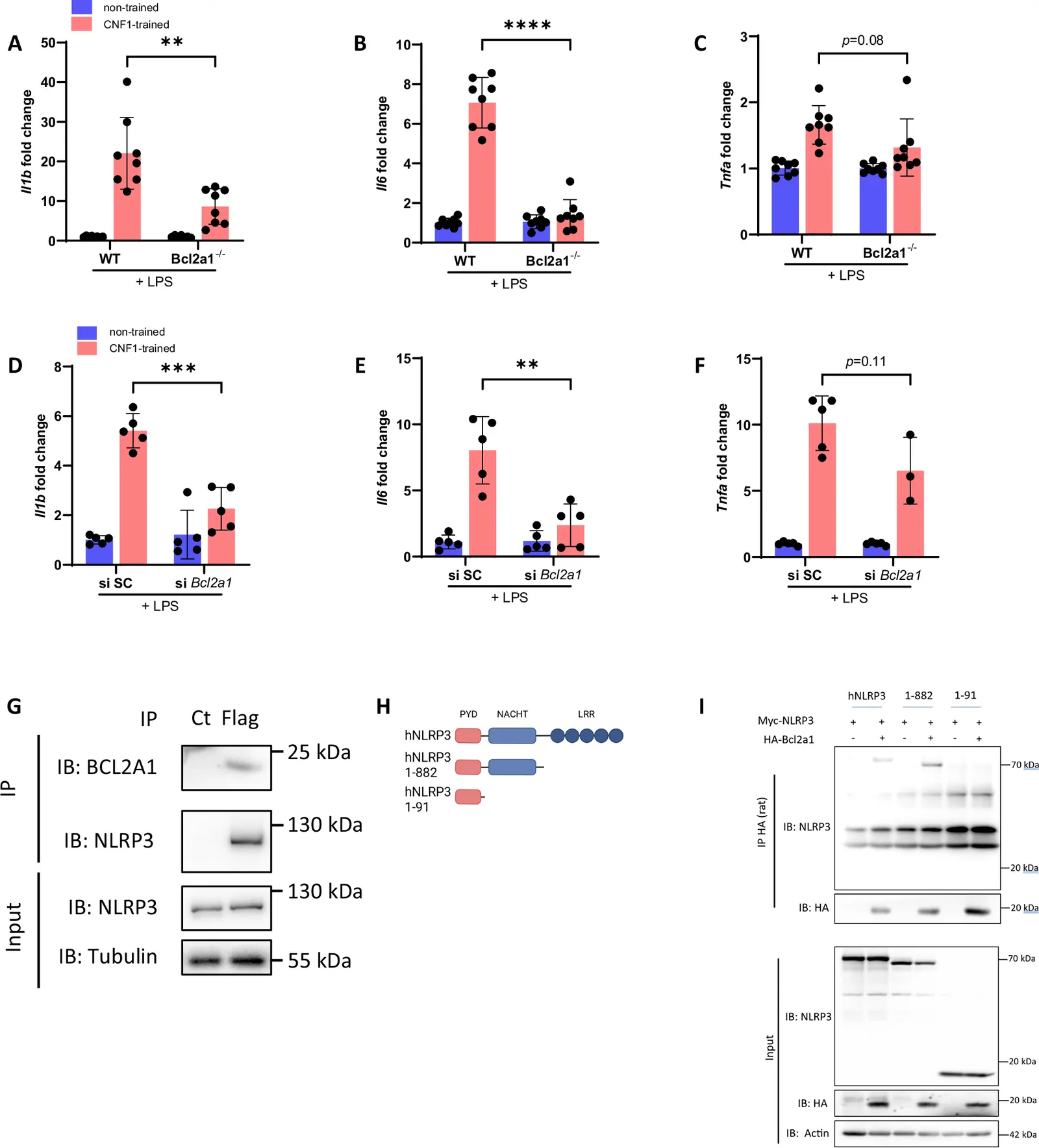
BCL2A1 role in CNF1-induced TI is conserved between murine and human macrophages and interacts with the NLRP3 inflammasome. **A**–**C** BMDM isolated from WT (*n* = 8) and Bcl2a1-knockout C57BL/6 J (*n* = 8) mice were trained with CNF1 (50 ng/mL) for 18 h before treatment with LPS for 24 h (10 ng/mL). Genes expression were analyzed using qPCR for *Il1b* ((**A**) *p* = 0.0036), *Il6* ((**B**) *p* < 0.0001) and *Tnfa* (**C**) Data are means ± SEM. Two-sided T-tests have been used for pairwise comparisons, with results considered statistically significant if *p* ≤ 0.05 (*p* ≤ 0.05: * - ≤ 0.01: ** - ≤ 0.001: *** - ≤ 0.0001: ****). Experiments were repeated three times. **D**–**F** Human monocyte-derived macrophages (hMDM) isolated from healthy donors were trained with CNF1 (50 ng/mL) for 18 h, transfected with siRNA targeting *Bcl2a1* (*n* = 5) or si scrambled (*n* = 5) for 72 h before treatment with LPS for 24 h (10 ng/mL). Genes expression were analyzed using qPCR for *Il1b* ((**D**) *p* = 0.0003), *Il6* ((**E**) *p* = 0.0043) and *Tnfa* (**F**). Data are means ± SEM. T-tests have been used for pairwise comparisons, with results considered statistically significant if *p* ≤ 0.05 (*p* ≤ 0.05: * - ≤ 0.01: ** - ≤ 0.001: *** - ≤ 0.0001: ****). Two-sided tests were used. Experiments were repeated three times. **G** iBMDM stably expressing Flag-NLRP3 were processed for immunoprecipitation. Cells were processed with anti-Flag-coated beads or control anti-HA-coated beads. Experiments were repeated three times with similar results. **H** Graphical representation of NLRP3 and the truncated mutant used. **I** HEK293T cells were transfected with plasmids encoding Myc-NLRP3 and HA-BCL2A1. Cell lysates were processed for anti-HA immunoprecipitation. Experiments were repeated four times with similar results.

To confirm the major role of BCL2A1 in the CNF1-triggered TI we used macrophages isolated from mice KO for *Bcl2a1*. First, we verified that the CNF1 toxin triggered Rac activation was not impaired in *Bcl2a1*^−/−^ macrophages which responded similarly to the wildtype macrophages to the CNF1 toxin treatment (Suppl. Fig. 2A, B). We then investigated the capacity of *Bcl2a1*^−/−^ macrophages to mount a CNF1-triggered TI. We measured a significant reduced expression of both IL-1β and IL-6 cytokines specifically in *Bcl2a1*^−/−^ macrophages (Fig. 4A, B). TNFα cytokine expression followed the same trend, although not significant (Fig. 4C). Next, we addressed the question of the conservation of this phenotype in human macrophages. To this aim, monocytes were sorted from peripheral blood mononuclear cells (PBMC) from healthy donors and were cultured and differentiated into macrophages using CSF-1. To determine the conservation of the role of BCL2A1 we used siRNA targeting *Bcl2a1* transcripts. Importantly, we were able to measure a conserved CNF1-triggered trained phenotype with an increased IL-1β, IL-6 and TNFα expression in human monocyte-derived macrophages (hMDM) that were significantly reduced in *Bcl2a1* knockdown condition (Fig. 4D, E). The same trend was observed for TNFα, even though it was not significant (Fig. 4C, F). Altogether our results showed a CNF1-induced TI that relies on the *Bcl2a1* gene expression in murine and human primary macrophages. BCL2A1 is known to play a critical role in regulating apoptotic cell death with important pro-survival functions whereas NLRP3 is a key mediator of pyroptotic cell death^21^. Since we showed that CNF1-induced TI relies on both BCL2A1 and NLRP3, we hypothesized that they may act through a common molecular mechanism. We therefore investigated the potential interaction between NLRP3 and BCL2A1. We first investigated this interaction in macrophages using iBMDM cells stably expressing Flag-NLRP3^22^. Using this system, we detected an interaction between NLRP3 and endogenous BCL2A1, indicating that these proteins associate closely enough to be co-immunoprecipitated (Fig. 4G). We next investigated which domain of NLRP3 mediates its interaction with BCL2A1 (Fig. 4H). Using an overexpression system in HEK293T cells to facilitate the detection of weak interactions, we found that HA-BCL2A1 co-immunoprecipitated with both full-length NLRP3 and the truncated NLRP3 (1–882) mutant lacking the LRR domain. These results indicate that the C-terminal LRR domain is not required for BCL2A1 binding (Fig. 4I). In contrast, we did not observe a co-immunoprecipitation between BCL2A1 and NLRP3 PYRIN domain (1-91) (Fig. 4I). These results indicated that the interaction occurs most probably via the NACHT domain. The NACHT domain being critical in regulating the NLRP3 activity, these results could explain the importance of BCL2A1 in regulating the NLRP3 inflammasome. Reinforcing this hypothesis, triggering the NLRP3 inflammasome by LPS plus CNF1 toxin treatment of *Bcl2a1*^−/−^ macrophages had a significant increase in IL-1β secretion whereas IL-6 secretion remained unchanged compared with wild-type macrophages (Suppl. Fig. 2C, D). Altogether, these results suggested BCL2A1 may act as a negative regulator of the NLRP3 inflammasome. We next tested whether this interaction was specific to NLRP3. To address this question, we tested other NLRs: human NLRP1, NLRP10 and Pyrin. Using the same experimental settings, we could not detect any interaction between BCL2A1 and the other NLRs tested (Suppl. Fig. 3).

### CNF1 toxin induces transient myeloid reprogramming and protects against lethal infection

**Fig. 5:**
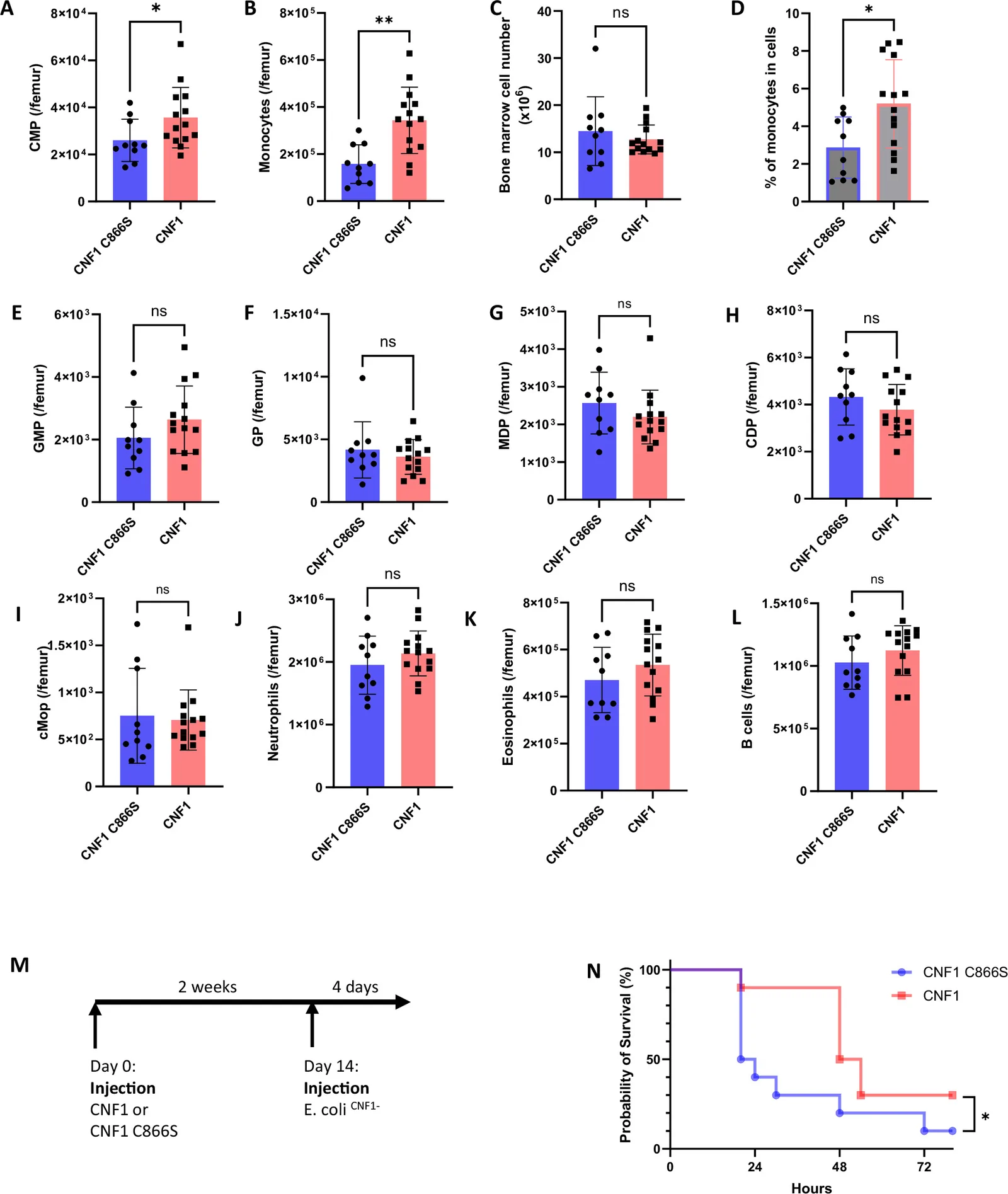
CNF1 toxin induces central TI and protects against sepsis. **(A - L)** WT C57BL/6 J mice were trained with intravenous injection of CNF1 (*n* = 14) (250 ng/kg) or its catalytic inactive mutant (CNF1 C866S) (*n* = 10) for 14 days before bone marrow cells were isolated and analyzed. (A) *p* = 0.0417, (B) *p* = 0.0015, (D) *p* = 0.0135. Data are means ± SEM. Two-sided Mann-Whitney tests have been used for pairwise comparisons, with results considered statistically significant if *p* ≤ 0.05 (*p*: ≤ 0.05: * - ≤ 0.01: **). **(M - N)** WT C57BL/6 J mice were trained with intravenous injection of WT CNF1 (*n* = 10) or its catalytic inactive mutant (CNF1C866S) (*n* = 10) (250 ng/kg) 14 days before intra peritoneal infection with UTI89 *E. coli* ^CNF1-^ strain at 10^6^ CFU. (N) *p* = 0.0317. T-tests have been used for pairwise comparisons, and Wilcoxon test otherwise with results considered statistically significant if p ≤ 0.05 (p: ≤ 0.05: * - ≤ 0.01: **). Two-sided tests were used.

Previous works have established the importance of myeloid progenitors and monocytes of the bone marrow for the TI in mice^23^. We thus hypothesized that the CNF1 triggered TI relies on monocytes from the bone marrow. To test this hypothesis in our model, mice were trained with injection of CNF1 toxin and myeloid progenitors were analyzed in the bone marrow after 14 days. Using this setting, we measured an increased number of common myeloid progenitors (CMP), which are oligopotent cells that can differentiate into granulocytes and macrophages, as well as monocytes in the bone marrow of mice injected with CNF1 compared to the control CNF1 catalytic inactive mutant (Fig. 5A, B). Although the total number of cells in the bone marrow was not significantly affected, we measured an increase in the percentage of monocytes (Fig. 5C, D and Suppl. Fig. 4). In contrast the other cells present in the bone marrow were not significantly affected (Fig. 5E–L). We observed this effect in monocytes, but not in closely related myeloid cells such as neutrophils and eosinophils, suggesting that TI likely impacts a specific monocyte progenitor rather than myelopoiesis in general. The increased number of CMP suggests that epigenetic and metabolic changes might happen in the bone marrow, allowing the generation of functionally adapted cells, such as the monocytes. Interestingly, these observations prompted us to extend our analysis to later time points (Suppl. Fig. 5). We found that the ex vivo trained immune phenotype, as assessed by cytokine expression of macrophages, was observed after one and 2 weeks but was no longer statistically significant at 3 and 4 weeks post-training, indicating that the cytokine component of CNF1-induced trained immunity is transient (Suppl. Fig. 5A–L). In contrast, changes in circulating immune cell populations appeared with a slight delay. Alterations in blood leukocyte composition became evident at 2 and 3 weeks after training but were no longer detectable by week 4 (Suppl. Fig. 5M, N). Together, these findings suggest that functional reprogramming of innate immune responses precedes the remodeling of circulating immune cell populations and that both processes are reversible over time.

To further address the physiological impact of CNF1-triggered TI, we investigated whether CNF1-trained mice were more resistant to sepsis triggered by the *E. coli* strain UTI89. To avoid a possible impact of antibodies directed against CNF1, we used a strain genetically deleted for the CNF1 gene (*E. coli* ^CNF1-^)^7,24^. We trained mice using CNF1 or the catalytically inactive CNF1 mutant 14 days before infection with the *E. coli* ^CNF1-^ (Fig. 5M). Importantly, mice injected with CNF1 prior to infection exhibited a significantly increased probability of survival compared with those injected with the catalytically inactive CNF1 mutant (Fig. 5N). Notably, 50% survival was observed at 20 h in control mice, whereas 50% survival was reached only at 48 h in CNF1-trained mice (Fig. 5N). This result indicates that the CNF1 toxin induces a protective trained immunity in vivo against sepsis.

## Discussion

Despite an exhaustive description of TI in various inflammatory contexts, how TI is regulated at the molecular level is still mostly unknown. Here, we show that TI is induced following the bacterial virulence factor sensing by the NLRP3 inflammasome. We first identified that TI was induced by treatment of mice with the CNF1 toxin in vivo, and next we set up an in vitro model that allowed us to characterize the molecular actors of this immune training. Using this system and following an unbiased screen, we unraveled BCL2A1 as a main regulator of TI, linking the the anti-apoptotic protein BCL2A1 to the NLRP3 inflammasome. While the genetic characterization and the description of the anti-apoptotic function of *Bcl2a1* dates back almost 30 years^21,25–27^, the immune role assigned to BCL2A1 has been challenged with the development of the *Bcl2a1* knockout mouse model^20,28,29^. The late development of the *Bcl2a1*^−/−^ mice is explained by the presence of this gene in 4 copies in the murine genome: the *Bcl2a1a*, *Bcl2a1b* and *Bcl2a1d* genes encode BCL2A1 family members, while *Bcl2a1c* is a pseudogene. Given the functional redundancy of the three BCL2A1 family members, finding them together in the top 20 genes overexpressed during training with the CNF1 toxin was expected. In contrast, the siRNA screen reveals the specific involvement of *Bcl2a1b*, which invalidation induced a significant loss of the training phenotype in macrophages, meaning that despite their redundancy, BCL2A1 family members may have functional differences.

The NLRP3 inflammasome activation was shown to be critical in the induction of TI induced by the western diet but little is known about the importance of inflammasome in the induction of TI during infection^16^. Here, we showed that the activation by a microbial toxin of the NLRP3 inflammasome induced the immune training phenotype revealed by a second stimulation by LPS. Interestingly, the activation of the NLRP3 inflammasome was previously investigated after an initial stimulation with β-glucan^30^. While β-glucan is the prototypical inducer of TI^12^, in this context β-glucan was shown to induce a decrease in the activation of the NLRP3 inflammasome on human monocytes from healthy donors but also from patients with cryopyrin-associated periodic syndrome (CAPS), an autoinflammatory condition caused by gain-of-function mutations in NLRP3^30^.

The NLRP3 inflammasome maturates both pro-IL-1β and pro-IL-18 into their biologically active forms, but in our results only recombinant IL-1β induced TI. If both cytokines have been associated with TI, the proofs on IL-18 as a potent TI inducer are scarce compared to IL-1β^15,31^. A recent report added further proof of IL-1β implication in TI through AIM2 (absent in melanoma 2) inflammasome activation, establishing IL-1β reprogramming of monocytes / macrophages responsible for the secondary comorbidities that follow a stroke^32^. Here, we expand the repertoire of inflammasome activators capable of inducing trained immunity by demonstrating that both the NLRP3 activators CNF1 and nigericin, as well as the NLRC4 activator T3SS needle protein, elicit this response. These findings further support the notion that inflammasomes are central regulators of trained immunity induced by diverse microbial stimuli.

There is a notable difference in the level of TI induction between CNF1 and Nigericin. Nigericin-induced TI shows higher levels of proinflammatory cytokines expression than with CNF1-induced TI. The secretion of IL-1β is different depending on whether Nigericin or CNF1 activates the NLRP3 inflammasome. While in BMDM CNF1 induces a secretion of IL-1β independent of gasdermin D (GSDMD)^7^, Nigericin induces the cleavage and the formation of GSDMD pores in the cell membrane through which IL-1β is secreted^33^. In vitro, less time is needed for Nigericin compared to CNF1 to produce similar quantities of IL-1β, suggesting that the kinetic of the Nigericin-induced IL-1β release is different from the CNF1-induced IL-1β release^7^. An explanation of the difference in amplitude of the Nigericin- and CNF1-induced trained immune response could be the difference in IL-1β secretion modality, but this hypothesis needs further investigation.

Another interesting aspect is related to our results describing a change of composition in the bone marrow with a transient increase of myeloid progenitor cells after injection with CNF1 toxin. This is in phase with what has been proposed previously and termed central trained immunity where epigenetic reprogramming and metabolic rewiring can affect bone marrow cells, leading to a subsequent production of trained innate immune cells^34^. This phenotype, combined with our mouse survival experiment indicating a protective effect of CNF1 injection, strengthens our conclusion that a trained immunity response is triggered by the sensing of this virulence factor.

As observed following sepsis or stroke, acute inflammatory insults can generate long-lasting disease trajectories associated with increased mortality and chronic comorbidities^32,35^. Thus, while CNF1-induced trained immunity may enhance resistance to secondary infections, persistent reprogramming of myeloid cells could also predispose to exaggerated responses to sterile inflammatory stimuli and promote inflammatory diseases. In this context, the transient nature of CNF1-induced trained immunity observed in our study may represent an adaptive feature rather than a limitation of this response. Bacterial virulence factors sensing may induce a self-limited form of trained immunity that provides temporary protection against recurrent infections while avoiding the risks associated with prolonged inflammatory reprogramming. The transient nature of CNF1-induced trained immunity we observed may therefore represent an important safeguard allowing a protective innate immune memory while minimizing the risk of long-term immunopathology. Such a mechanism would be particularly well suited to infections caused by extracellular bacterial pathogens, where a rapid but reversible enhancement of innate immunity may be sufficient to improve host defense without compromising immune homeostasis. Addressing this possibility will require dedicated clinical studies.

In conclusion, we have identified a major role for TI induced in response to bacterial virulence factors sensing by the NLRP3 inflammasome. We have discovered that the importance of BCL2A1 protein in regulating TI in this context and, using a model of bacterial systemic infection, we demonstrated TI was protective against deadly *E. coli* sepsis. Altogether, our model suggests that sensing of virulence factors by the NLRP3 inflammasome is sufficient to trigger innate immune central memory, providing mice with protection against *E. coli* sepsis. Importantly, the transient nature of this response suggests that bacterial toxins promote a reversible form of trained immunity, balancing short-term protection against infection with preservation of long-term immune homeostasis.

## Methods

### Ethics statement

This study was carried out in strict accordance with the guidelines of the Council of the European Union (Directive 86/609/EEC) regarding the protection of animals used for experimental and other scientific purposes. The protocol was approved by the Institutional Animal Care and Use Committee on the Ethics of Animal Experiments of Nice, France (APAFIS#18322-20181218099427035 v2 and APAFIS#24906-2020031614223228 v2). The study was conducted in accordance with French legislation and the principles of the Declaration of Helsinki with the informed consent obtained from the blood donors. Human blood from healthy donors was obtained from the Etablissement Français du Sang (N13002).

### Bacterial toxins and strains

Recombinant WT CNF1 and its catalytically inactive form (CNF1 C866S) were produced and purified as previously reported^36,37^. The *E. coli* OneShot strain carrying the pCRIIcnf1 plasmid was grown overnight at 37 °C in LB medium. Bacteria were lysed in PBS using a French press. Bacterial lysate was centrifuged at 10,000 *g*, and the supernatant was precipitated with an equal volume of saturated ammonium sulfate for 5–8 h at 4 °C. The precipitate was dialyzed against TN buffer (25 mM Tris-HCl, pH 7.4, 50 mM NaCl), overnight at 4 °C and applied on a DEAE fast flow column (11×1.5 cm; Pharmacia Biotech) equilibrated with TN buffer. The column was washed 200 min at 1 mL/min flow rate with TN buffer. CNF1 was eluted at 200 mM NaCl (gradient of 50–300 mM NaCl, 100 min). The peak of eluted protein was determined by measure of the absorbance at 280 nm. The fractions containing CNF1 were pooled, dialyzed against TN buffer overnight, and applied on a Superdex 75 column at 0.3 mL/min (30×1 cm, Pharmacia Biotech). The fractions containing CNF1 were pooled, concentrated on a 30-kDa cutoff vivaspin (Vivascience) and applied on a monoQ column (5×0.5 cm, Pharmacia Biotech). The column was washed 30 min at 1 mL/min with TN buffer and CNF1 was eluted at 350 mM NaCl (gradient of 50–400 mM NaCl, 20 min). The level of CNF1 purification is assessed by SDS-PAGE followed by Coomassie brilliant blue staining. The recombinant proteins were passed through a polymyxin B column (Affinity Detoxi-Gel, Pierce). The removal of endotoxin was verified using a colorimetric limulus amebocyte lysate (LAL) assay (LAL QCL-1000, Cambrex). Each stock of the CNF1 preparation (2 mg/mL) was shown to contain less than 0.5 endotoxin units per mL.

The *E. coli* UTI89 clinical isolate was originally obtained from a patient with cystitis^38^ and the isogenic UTI89 ΔCNF1 ΔHlyα streptomycin-resistant strain generation and culture conditions were previously described^24^. For the infections, a 1/100 dilution of an overnight culture was inoculated and grown up to an optical density at 600 nm of 1.2 using a Luria-Bertani (LB) medium supplemented with streptomycin (200 μg/mL). Bacteria were collected by centrifugation and washed twice in PBS before dilution in PBS to obtain the desired bacterial concentrations for the mouse infection experiments.

### Mouse model of trained immunity

Six-week-old female C57Bl/6j WT mice (Janvier Labs) were injected intravenously with CNF1 toxin at 250 ng/kg in PBS. Two weeks after injection, mice were sacrificed by cervical dislocation and spleens were extracted for splenic macrophage isolation. Spleens were grinded with a syringe plunger over a 70 µm filter in a small Petri dish containing RPMI 1640 GlutaMax medium (Life Technologies) with 10 % (vol/vol) heat-inactivated fetal bovine serum (FBS) (Biowest) and 50 μg/mL gentamicin (Life Technologies) and the cell suspension was incubated at 37 °C with an atmosphere containing 5 % CO_2_. After 3 h of incubation, cells were washed 3 times with warm PBS and incubated for 7 days in culture medium supplemented with 100 ng/mL of M-CSF (Miltenyi Biotec). On day 8, splenic macrophages were stimulated with culture medium only (negative control) or 10 ng/mL of ultrapure LPS (InvivoGen). After 24 hours, cells were lysed in Trizol and processed for RNA extraction, supernatants were collected and stored at −20 °C until cytokine measurement.

For *E. coli* triggered sepsis experiments, six-week-old female C57Bl/6j WT mice (Janvier Labs) were injected intravenously with CNF1 toxin at 250 ng/kg. 14 days after injection, mice were injected intraperitoneally with 10^6^ colony-forming units (CFU) of *E. coli* and the determination survival was monitored as previously described^24^. Mice were housed with their littermates and kept under a regular 12h-12h light-dark cycle at room temperature (20-25 °C) and a relative humidity of 50-70 %. Food and water were available *ad libitum*. Experiments were performed under pathogen-free conditions. Sample size was determined using G*Power software.

### Primary cell extraction and culture

BMDM were extracted from the femurs of WT and KO C57BL/6 J mice (aged 6-10 weeks) as indicated in the legends. Bone marrow cells were cultured in RPMI 1640 GlutaMax medium (Life Technologies) supplemented with 100 ng/mL M-CSF (premium grade, Miltenyi Biotec), 10 % (vol/vol) heat-inactivated FBS (Biowest) and 50 μg/mL gentamycin (Life Technologies) at 37 °C in an atmosphere containing 5 % CO_2_. The cells were seeded at a concentration of 400.000 cells per well in a 12-well plate.

Buffy coats from volunteers were ordered from the French blood establishment. PBMC were isolated using Ficoll (Cytiva), and red cells were lysed using lysing buffer (BD Biosciences). Then, monocytes were purified using anti-CD14 microbeads (Miltenyi) and autoMACS Pro Separator (Miltenyi) according to the manufacturer’s instructions. Monocytes were cultivated in RPMI 1640 GlutaMax medium (Life Technologies) supplemented with 10 % (vol/vol) heat-inactivated FBS (Biowest) and 50 μg/mL gentamycin (Life Technologies) at 37 °C in an atmosphere containing 5 % CO_2_. Macrophage differentiation was induced by addition of CSF-1 (100 ng/mL, Miltenyi) into the culture medium.

### Cell model of trained immunity

The cell model of TI was derived from Christ et al.^16^. Briefly, after 48 to 72 hours of differentiation, BMDM or human monocytes were incubated (day 0) with culture medium only (negative control) or 50 ng/mL CNF1 for 18 hours. Cells were washed with warm PBS and incubated for 5 days in their respective culture medium. On day 6, the cells were re-stimulated with culture medium only (negative control) or 10 ng/mL ultrapure LPS (InvivoGen). After 24 hours, cells were lysed in Trizol and processed for RNA extraction, supernatants were collected and stored at −20 °C until cytokine measurement. For training inductions, cells were treated 18 h with CNF1 50 nM (except specified otherwise), Nigericin 100 nM (InvivoGen) or mouse recombinant IL-1β or IL-18 (Bio-Techne).

### Transfection

siRNAs were transfected into mouse BMDM or human monocyte-derived macrophages (hMDM) using Lipofectamine RNAiMAX Reagent, according to manufacturer’s instructions (ThermoFisher Scientific). 500.000 hMDM or 300.000 BMDM were seeded in a 12-well plate for 3 or 5 days respectively, transfected with siRNA at a final concentration of 50 nM, and incubated for 72 hours before trained immunity assessment. ON-Target plus SMARTpool siRNA and ON-Target plus non-targeting control pool siRNA were acquired from Horizon Discovery. Cells were transfected as indicated in the figure legends with siRNA targeting Human *Bcl2a1* (L-003306-00), or non-targeting control siRNA (D-001810-10). For the siRNA screen, the BMDM were transfected with siRNA targeting Mouse *Adgrg6* (L-066274-00), *Akap6* (L-066387-01), *Akap9* (L-041065-01), *Ass1* (L-061969-02), *Bcl2a1a* (L-062467-01), *Bcl2a1b* (L-043036-00), *Bcl2a1d* (L-043046-00), *Cd300lf* (L-055009-01), *Cd33* (L-047462-01), *Cish* (L-062498-00), *Ddit3* (L-062068-00), *Denr* (L-040647-01), *Gfpt1* (L-062877-01), *Il12b* (L-042612-01), *Il1a* (L-042612-01), *Il6* (L-043739-00), *Kcnq1ot1* (R-174157-00), *Klf9* (L-059033-01), *Lilrb4a* (L-042362-01), *Ly6c2* (L-168566-00), *Macf1* (L-050656-00), *Matn2* (L-046970-02), *Mdn1* (L-167414-00), *Mmp12* (L-047445-01), *Mtap* (L-063657-01), *Nes* (L-057300-01), *Pde10a* (L-044706-00), *Pdia4* (L-048826-01), *Pdia5* (L-055399-01), *Prkg1* (L-048829-00), *Slc7a1* (L-042922-01), *Sqstm1* (L-047628-01), *Uck2* (L-047637-00), *Wdfy4* (L-068186-01), *Wnt6* (L-042137-01) and *Zfyve21* (L-056556-01).

### RNA isolation and RT-PCR

Total RNA was extracted using TRIzol (ThermoFisher Scientific), according to the manufacturer’s instructions, and quantified using a BioTek Synergy 2 plate reader. cDNA was synthesized from 500 ng of RNA using High Capacity cDNA Reverse Transcription Kit (Applied Biosystems). Relative expression was determined using Power SYBR Green (Applied Biosystems) on a StepOne PLUS RT-PCR machine (Applied Biosystems). Fold changes in expression were calculated by the ΔΔCt method^39^ using *Gusb* (mouse) or *Actin* (human) as endogenous controls. At least three independent replicate samples were prepared for qPCR analysis, and statistical test was performed on ΔCt values. The primer pairs used can be found in Suppl. Table 1.

### RNA- and ATAC-sequencing

BMDM were detached from their support and then cryopreserved for shipment to Active Motif, which performed ATAC-seq and RNA-seq analyses. ATAC-seq was performed on BMDM harvested on day 6, previously trained with culture medium (negative control) or 50 ng/mL CNF1 for 18 h. RNA-seq was performed on BMDMs that received the same treatment as for ATAC-seq and harvested on day 7, after being stimulated with 10 ng/mL LPS for 24 h.

### Antibodies and ELISA assays

The following antibodies were used in this study: rabbit anti-IL-1β (GTX74034, Genetex, 1/1000), mouse anti-Rac (clone 102/Rac1, BD Biosciences, 1/1000), goat anti-Rac2 (ab2244, Abcam, 1/1000), mouse anti-NLRP3 (clone Cryo-2, Adipogen, 1/1000), rabbit anti-Pak1 (2602, CST, 1/1000), rabbit anti-Pak2 (2608, CST, 1/1000), mouse anti-β-actin (AC-74, Sigma-Aldrich at 1/2000). Cytokine secretion was determined by ELISA using the mouse Quantikine ELISA kit for mouse IL-6 and IL-1β (R&D Systems) according to the manufacturer’s instructions.

### Immunoprecipitation

HEK293T cells (ATCC **CRL-3216)** were transfected using Lipofectamine 2000 (Life Technologies) with pCDNA3 plasmids encoding Myc–NLRP3 and HA-BCL2A1. Sixteen hours post transfection, cells were lysed at 4 °C using a lysis buffer (Tris 50 mM pH 7,5, NaCl 150 mM, MgCl2 10 mM, Nonodet P40 1%). Immunoprecipitation was performed using 2 μg of anti-HA antibodies (Roche clone 3F10). The expression of NLRP3 and BCL2A1 was monitored in the cell lysate as well as in the immunoprecipitated fraction by immunoblotting anti-HA (901515, Biolegend, 1/1000) and anti-NLRP3 (AG-20B-0014-C100, Coger, 1/1000). NLRP1 Immunoprecipitation experiments were done using anti-NLRP1 (679802, Biolegend, 1/1000). Equal amount of proteins engaged in the immunoprecipitation was confirmed by immunoblotting anti-β-actin (3700S, Cell Signalling technology, 1/1000).

iBMDM (kindly provided by Dr. Bénédicte Py) stably expressing Flag-NLRP3 (NG5) were lysed at 4 °C using a lysis buffer (Tris 50 mM pH 7,5, NaCl 150 mM, MgCl2 10 mM, Nonodet P40 1%). Immunoprecipitation was performed using 20 µL of anti-Flag-coated beads. The expression of NLRP3 and BCL2A1 was monitored in the cell lysate as well as in the immunoprecipitated fraction by immunoblotting anti-Flag (F1804, Sigma-Aldrich, 1/1000) and anti-BCL2A1 (kindly provided by Marco J. Herold, Melbourne, Australia, 1/100). Equal amount of proteins engaged in the immunoprecipitation was confirmed by immunoblotting anti-tubulin (T4026, Sigma, 1/1000). Blots analyses were done using Fiji v2.1.0 by ImageJ.

### Cytokine measurements

Cytokine production was assessed in supernatants using commercial ELISA kits for mouse IL-1β, IL-6 and TNFα (R&D systems, MN, USA), in accordance with the manufacturer’s instructions.

### Flow cytometry analysis

Bone marrow cells were extracted as previously described. Red blood cells were lysed using BD Pharm Lyse buffer (BD Biosciences) according to the manufacturer’s instructions. Cell-surface markers were stained in Flow Cytometry Buffer (PBS; 1% BSA; 2 mM EDTA) for 30 min in the dark at 4 °C using the following recombinant antibodies (1/200; BioLegend):, CD115-BV711 (clone AFS98), CD117-BV510 (clone ACK2), CD11b-BV750 (clone M1/70),, CD135-APC (clone A2F10), CD16/32-PercP 5.5 (clone 93), CD170-PE (clone S17007L), CD19-BV570 (clone 6D5), CD34-PE Cy5 (clone MEC14.7) (1/100), CD45-APC Fire 810 (clone 30-F11), IA/IE-Pacific Blue (clone M5/114.15.2), Ly6C-BV605 (clone HK1.4), Ly6G-BV785 (clone 1A8). Cells were fixed with 4% paraformaldehyde for 10 min at room temperature. Flow cytometry was performed using an Aurora flow cytometer from Cytek. Collected data were then analyzed with FlowJo and GraphPad Prism softwares. Bone marrow gating strategy used in this study is represented in Suppl. Fig. 3.

### Statistical analyses

Statistical analyses were performed using GraphPad Prism software. Pairwise comparisons were performed using two-tailed Student’s t-tests, and group comparisons were performed using two-way ANOVA.

### Reporting summary

Further information on research design is available in the Nature Portfolio Reporting Summary linked to this article.

## Supplementary information

Supplementary Information

reporting summary

Transparent Peer Review file

## Source data

source data

## Data Availability

All data supporting the findings of this study are available within the article and its supplementary information or from the corresponding author upon reasonable request. The ATACSeq data and RNASeq data generated in this study and shown in Fig. 3 have been deposited in the GEO database. ATACSeq data (GSE337610) are available using the following link https://www.ncbi.nlm.nih.gov/geo/query/acc.cgi?acc=GSE337610. RNASeq data (GSE337763) are available using the following link https://www.ncbi.nlm.nih.gov/geo/query/acc.cgi?acc=GSE337763. Source data are provided with this paper.
