# Supplementary material for: Bacterial toxin sensing induces transient inflammasome-dependent innate immune memory that improves survival during sepsis
Source: Nat Commun. 2026 Oct 8;17:11460. doi: 10.1038/s41467-026-78499-5 (PMC13740286; doi:10.1038/s41467-026-78499-5)

Supplementary Information

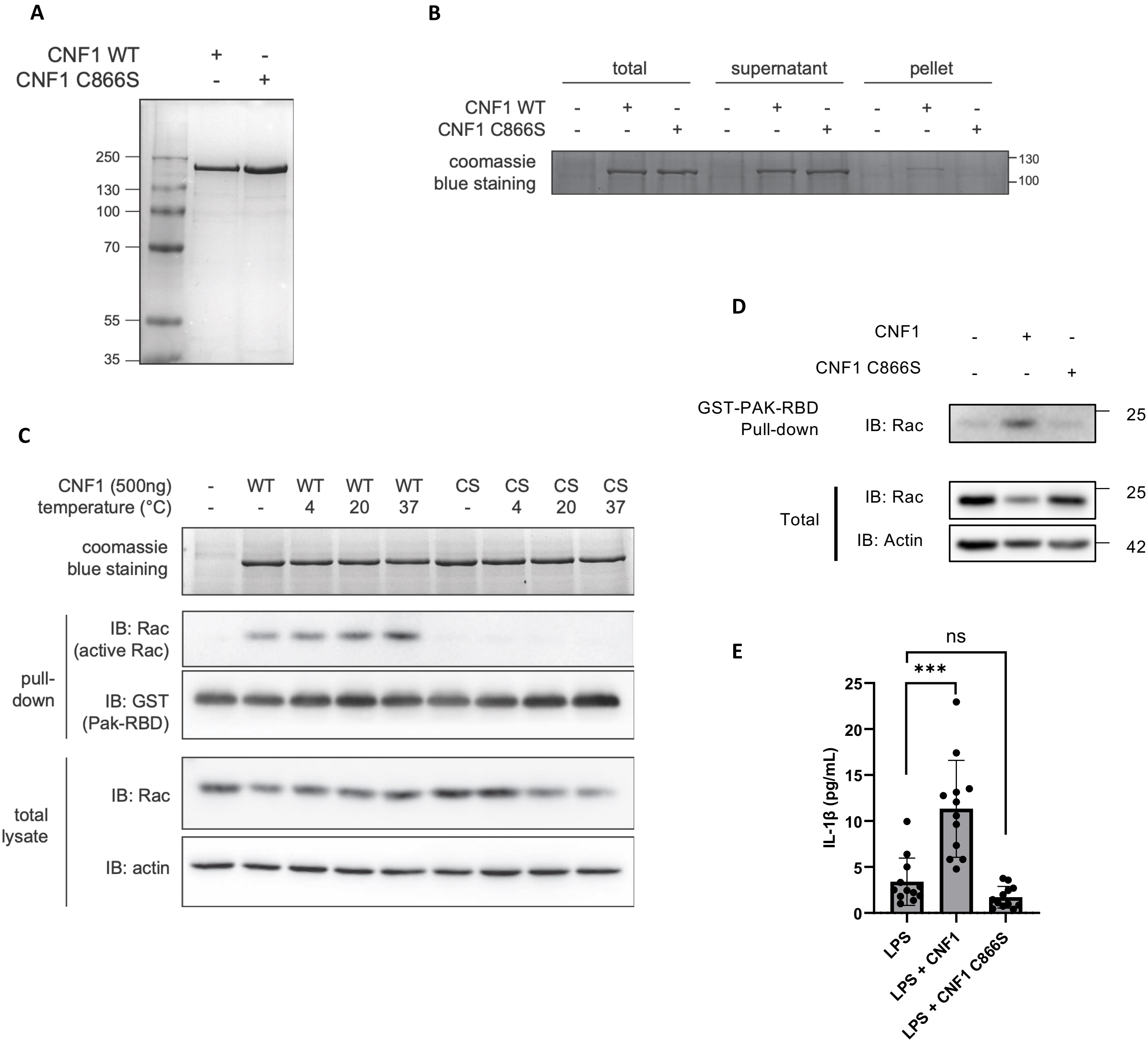

Supplementary Figure 1: Catalytic inactive mutant CNF1 C866S does not activate the NLRP3 inflammasome pathway

A. Comparison CNF1 WT and C866S purity. 500ng of CNF1 WT or catalytic inactive C866S were analyzed by SDS–PAGE followed by Coomassie blue staining. **B.** Comparison CNF1 WT and C866S solubility. One microgram of CNF1 WT or C866S was resuspended in 40 µL of cell media (RPMI, glutamax, pen-strep, FBS 2%). Half of the sample (20 µL) was kept as the total fraction, whereas the remaining 20 µL was centrifuged at 15,000 × g for 15 min at 4°C to separate supernatant and pellet fractions. The total, supernatant, and pellet fractions were all analyzed by SDS–PAGE followed by Coomassie blue staining. **C.** Comparison CNF1 WT and C866S stability. One microgram of CNF1 WT and C866S (CS) was incubated for 24 hours at the indicated temperature. For each condition, 500 ng were used to intoxicate HEK293T cells, and the remaining 500 ng were analyzed by SDS–PAGE followed by Coomassie blue staining. Toxin activity was assessed by immunoblot analysis of endogenous Rac (IB: Rac) and GST–Pak-RBD pull-downs (IB: Rac-GTP), from HEK293T cells. **D-E**. WT BMDMs were treated with LPS (100 ng/mL), LPS and CNF1 toxin (500 ng/mL) or LPS and CNF1 C866S (500 ng/mL) for 8h before analysis using a GST–PAK-RBD pull down assay (D); Supernatants were analyzed using enzyme-linked immunosorbent assay (ELISA) for IL-1ß (E). (E) n=12 Data are means ± SEM. Two-sided T-tests have been used for pairwise comparisons, with results considered statistically significant if $p \leq 0.05$ (p: ≤ 0.05 : * - ≤ 0.01 : ** - ≤ 0.001 : *** - ≤ 0.0001 : ****). On the graph *** corresponds to p=0.0003.

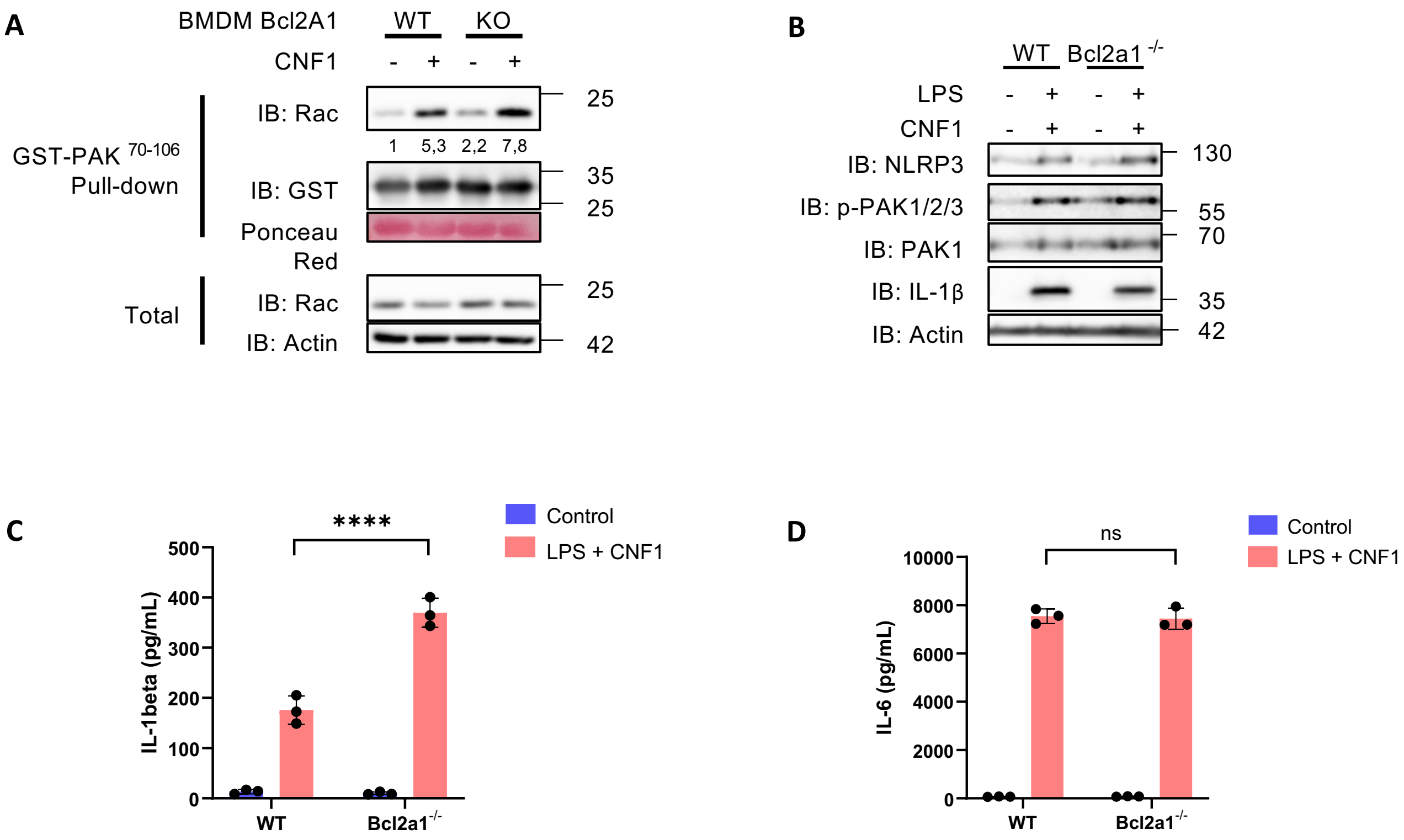

Supplementary Figure 2: Bcl2a1$^{-/-}$ macrophages does not show differences in CFN1 toxin-induced RacGTPase activation but an increase in IL-1β secretion

A. WT and Bcl2a1 $^{-/-}$ BMDMs were treated with CNF1 toxin (500 ng/mL) for 3h before analysis using a GST–PAK-RBD pull down assay. Rac2 associated with the GST–PAK-RBD beads is indicated as Rac2–GTP. **B.** WT and Bcl2a1 $^{-/-}$ BMDMs were treated with LPS (100 ng/mL) and CNF1 toxin (500 ng/mL) for 8h before the cell lysates were collected and analyzed using immunoblotting. **C-D.** WT and Bcl2a1 $^{-/-}$ BMDMs were treated with both LPS (100 ng/mL) and CNF1 toxin (500 ng/mL) for 8h before secreted levels of IL-1β and IL-6 were assessed in the medium by Elisa. (C) n=3, **** corresponds to $p<0.0001$. (D) n=3. Data are means ± SEM. Two-sided T-tests have been used for pairwise comparisons, with results considered statistically significant if $p \leq 0.05$ (p: ≤ 0.05 : * - ≤ 0.01 : ** - ≤ 0.001 : *** - ≤ 0.0001 : ****). Experiments were repeated three times.

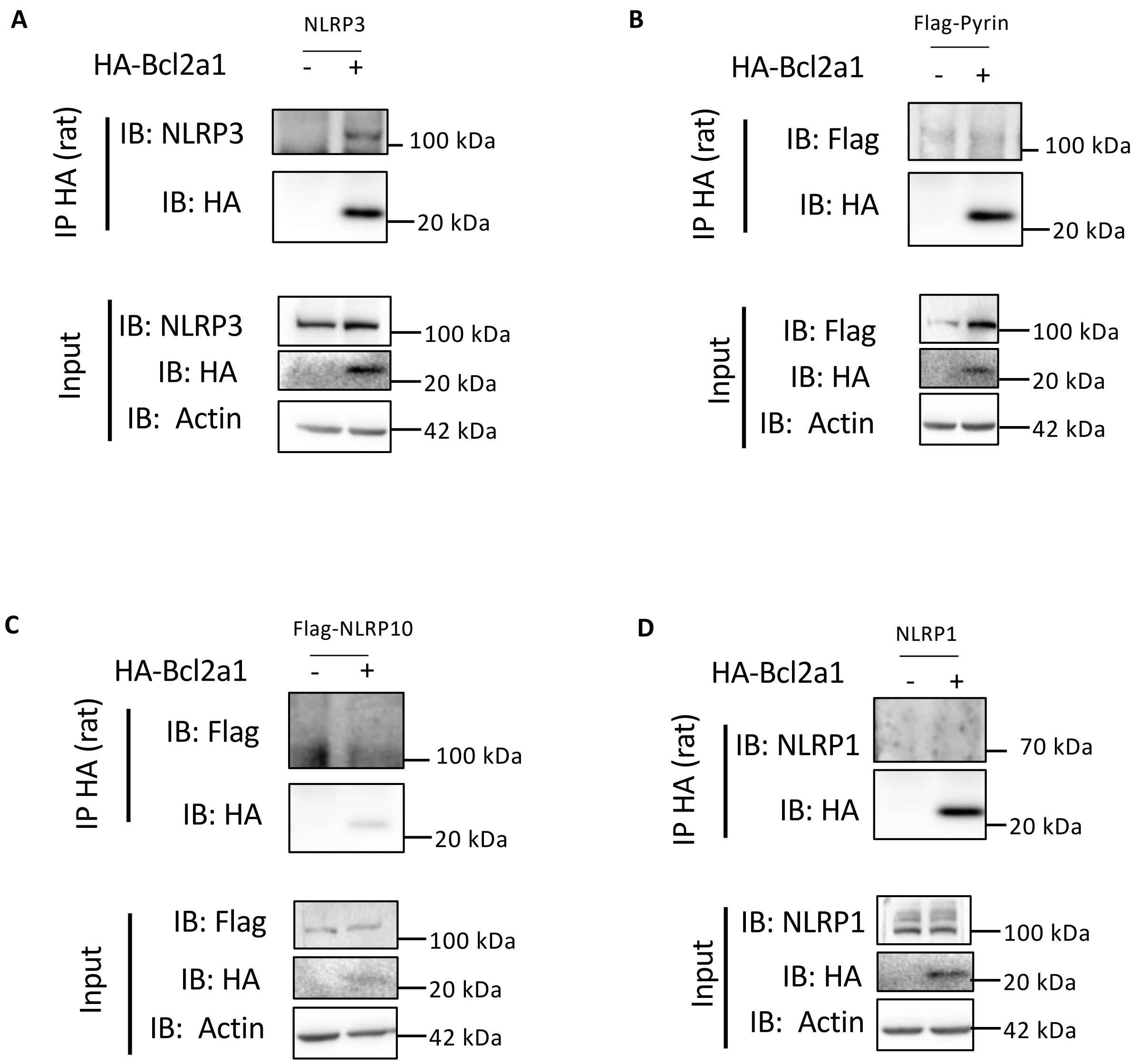

Supplementary Figure 3: BCL2A1 interact with NLRP3 but not with Pyrin, NLRP1 or NLRP10.

(A - D) HEK293T cells were transfected with plasmids encoding HA tagged human Bcl2a1 and Flag-tagged human Pyrin, or NLRP3, or Flag-NLRP10, or NLRP1. Cell lysates were processed for anti-HA immunoprecipitation (IP). Experiments were repeated three times.

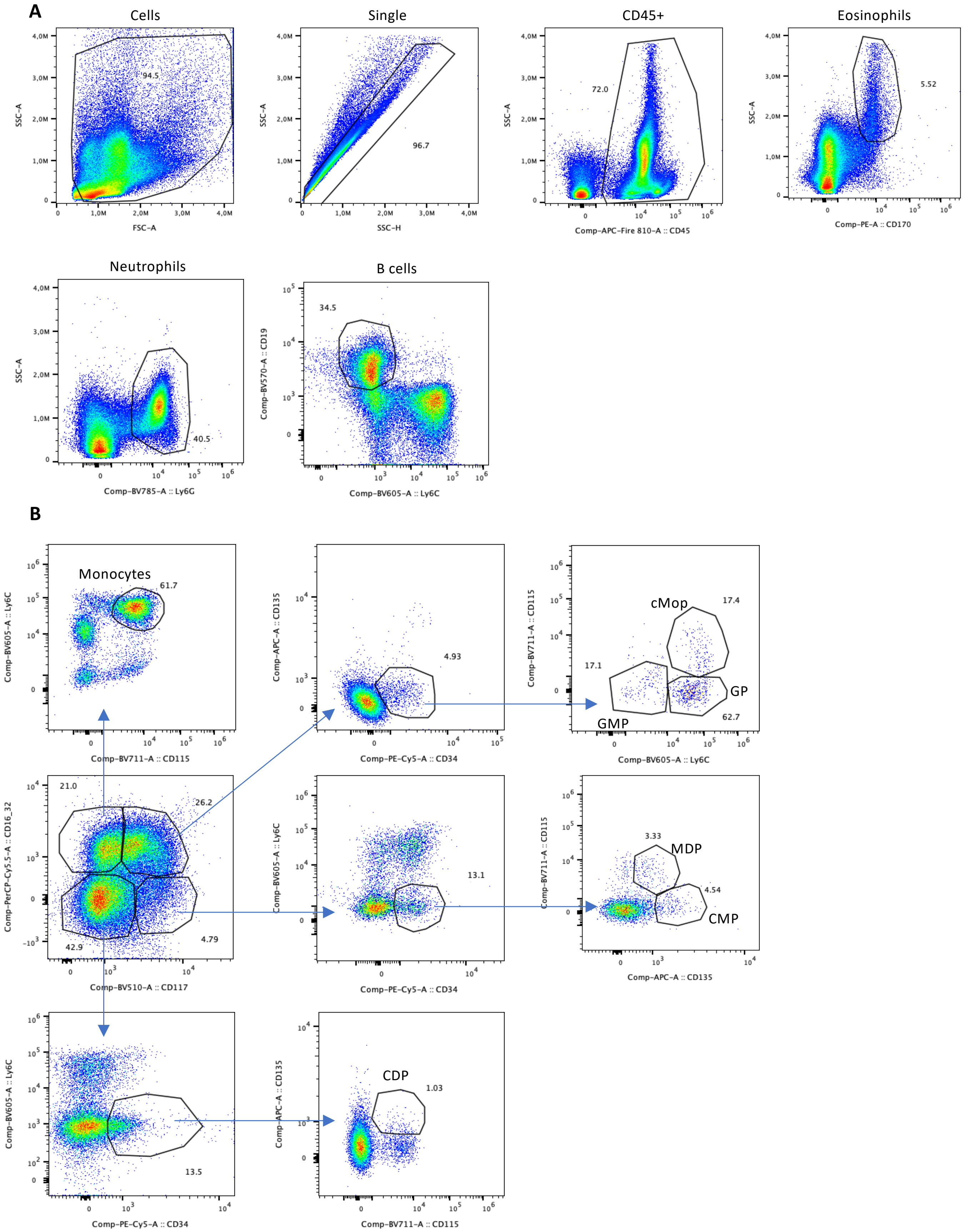

Supplementary Figure 4: Gating strategy of bone marrow immune cell populations

Flow cytometry analysis of cell populations in murine bone marrow tissue. **A**. Gate for nucleated cells based on the size and complexity of the event (FCS-A and SSC-A, respectively). Nucleated cells were further plotted in SSC-A and SSC-H to gate single cells and exclude doublets. From the single cell gate, leukocytes were defined as CD45+ and bone marrow immune cell subpopulations were gated as shown above. **B**. Myeloid innate immune cell progenitors were plotted as shown above.

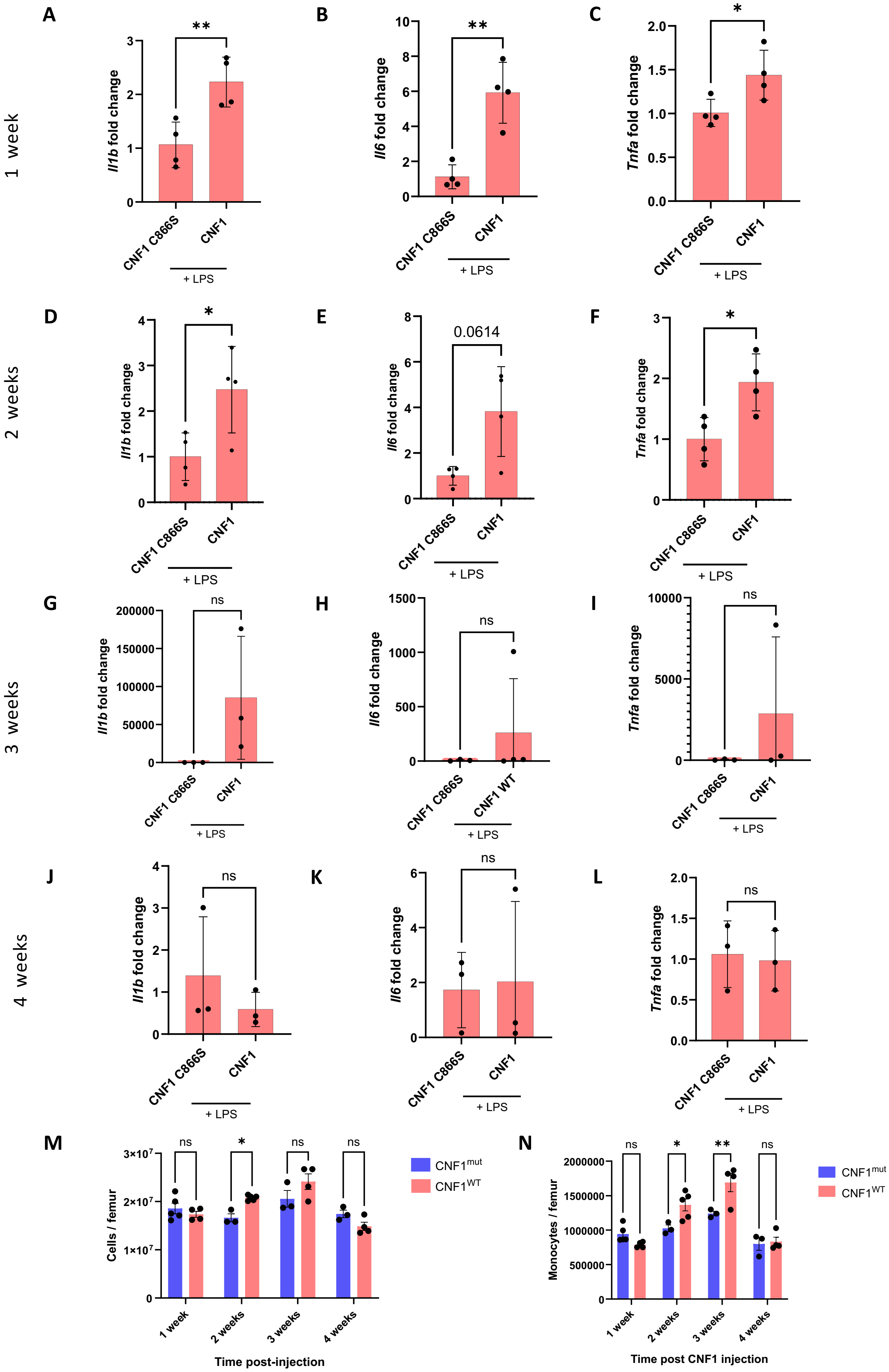
A
B
C
D
E
F
G
H
I
J
K
L
M
N
1 week
2 weeks
3 weeks
4 weeks
Il1b fold change
Il6 fold change
Tnfa fold change
CNF1 C866S
CNF1
CNF1 WT
+ LPS
**
*
ns
0.0614
Cells / femur
Monocytes / femur
Time post-injection
Time post CNF1 injection
CNF1mut
CNF1WT

Supplementary Figure 5: The CNF1-triggred trained immune memory is transiant

A-L WT C57BL/6J mice were trained with intravenous injection of WT CNF1 or its catalytic inactive mutant ($CNF1^{C866S}$) (250 ng/kg) one week (A, B, C), or two weeks (D, E, F), or three weeks (G, H, I) or four weeks (J, K, L) before splenic macrophages were isolated, cultured then treated with LPS for 24 h (10 ng/mL). Gene expression were analyzed using qPCR for *Il1b* (A, D, G, J), *Il6* (B, E, H, K) and *Tnfa* (C, F, I, L), expressed in fold change. A-F n=4. G-L n=3. A p=0.0099, B p=0.0021, C p=0.0379, D p=0.0452, F p=0.0210. Data are means ± SEM. Two-sided T-tests have been used for pairwise comparisons, with results considered statistically significant if $p \leq 0.05$ (*p*: ≤ 0.05 : * - ≤ 0.01 : ** - ≤ 0.001 : *** - ≤ 0.0001 : ****). **M-N** WT C57BL/6J mice were trained with intravenous injection of CNF1 or its catalytic inactive mutant ($CNF1^{mut}$) (250 ng/kg) for one week, two weeks, three weeks or four weeks before bone marrow cells were isolated and analyzed. N=4 M p=0.0422, N * corresponds to p=0.0247 and ** corresponds to p=0.0036. Data are means ± SEM. Two-Way ANOVA-tests have been used for pairwise comparisons, with results considered statistically significant if $p \leq 0.05$ (*p*: ≤ 0.05 : * - ≤ 0.01 : ** - ≤ 0.001 : *** - ≤ 0.0001 : ****).

SPECIES	GENE	FORWARD PRIMER	REVERSE PRIMER
**Human**	Actin	CTTCCTGGGCATGGAGTC	GGATGTCCACGTCACACTTC
**Mouse**	Gusb	GATTCAGATATCCGAGGGAAAGG	CCAACGGAGCAGGTTGAAAT
**Human**	Il1b	CTAAACAGATGAAGTGCTCC	GGTCATTCTCCTGGAAGG
**Mouse**	Il1b	GGATGATGATGATAACCTGC	CATGGAGAATATCACTTGTTGG
**Human**	Il6	GCAGAAAAAGGCAAAGAATC	CTACATTTGCCGAAGAGC
**Mouse**	Il6	AAGAAATGATGGATGCTACC	GAGTTTCTGTATCTCTCTGAAG
**Human**	Tnfa	AGGCAGTCAGATCATCTTC	TTATCTCTCAGCTCCACG
**Mouse**	Tnfa	CTATGTCTCAGCCTCTTCTC	CATTTGGGAACTTCTCATCC

Supplementary Table 1: Primer pairs used in the gene expression analysis

Supplementary Figure 1

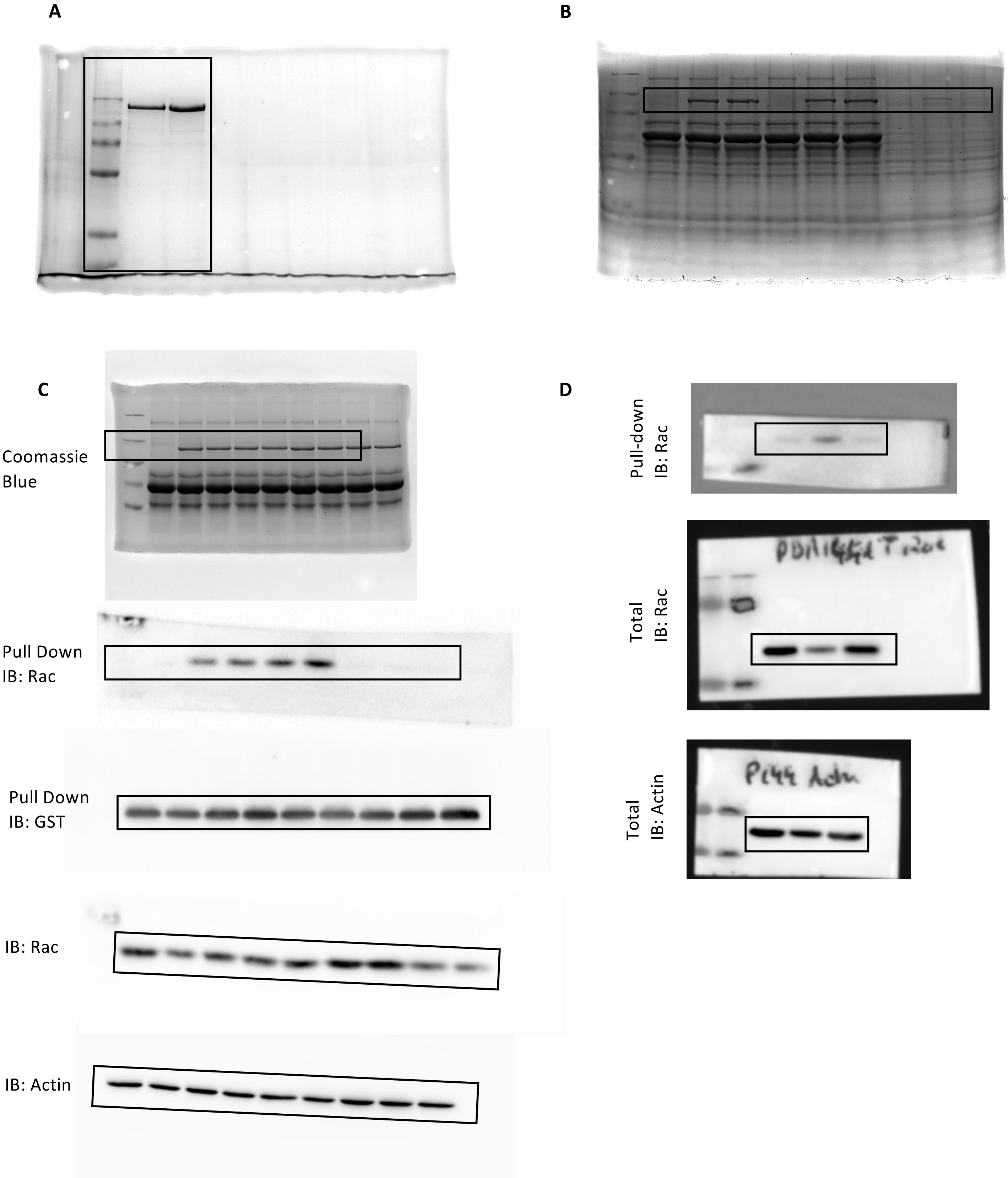

Supplementary Figure 2

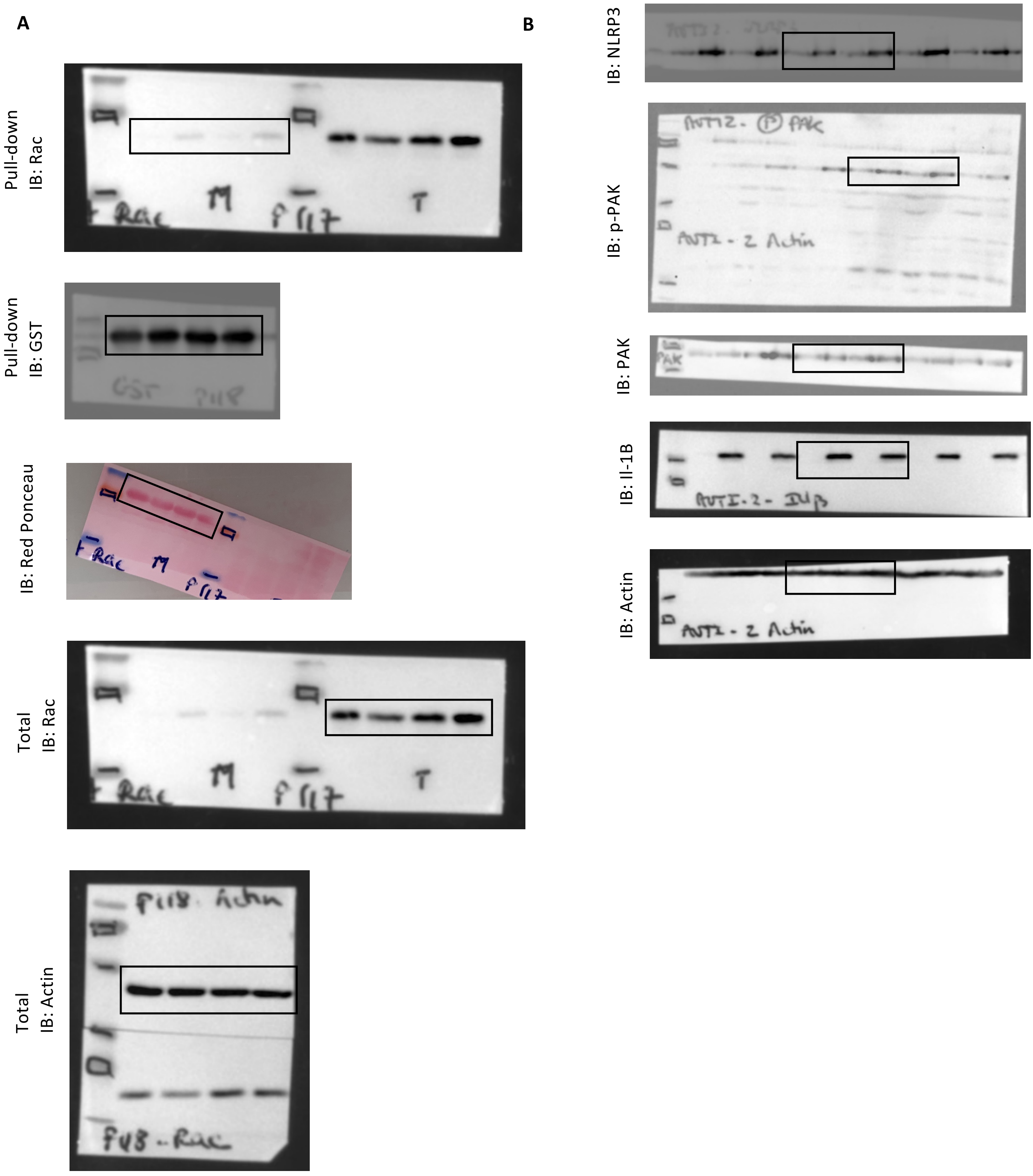

Supplementary Figure 3

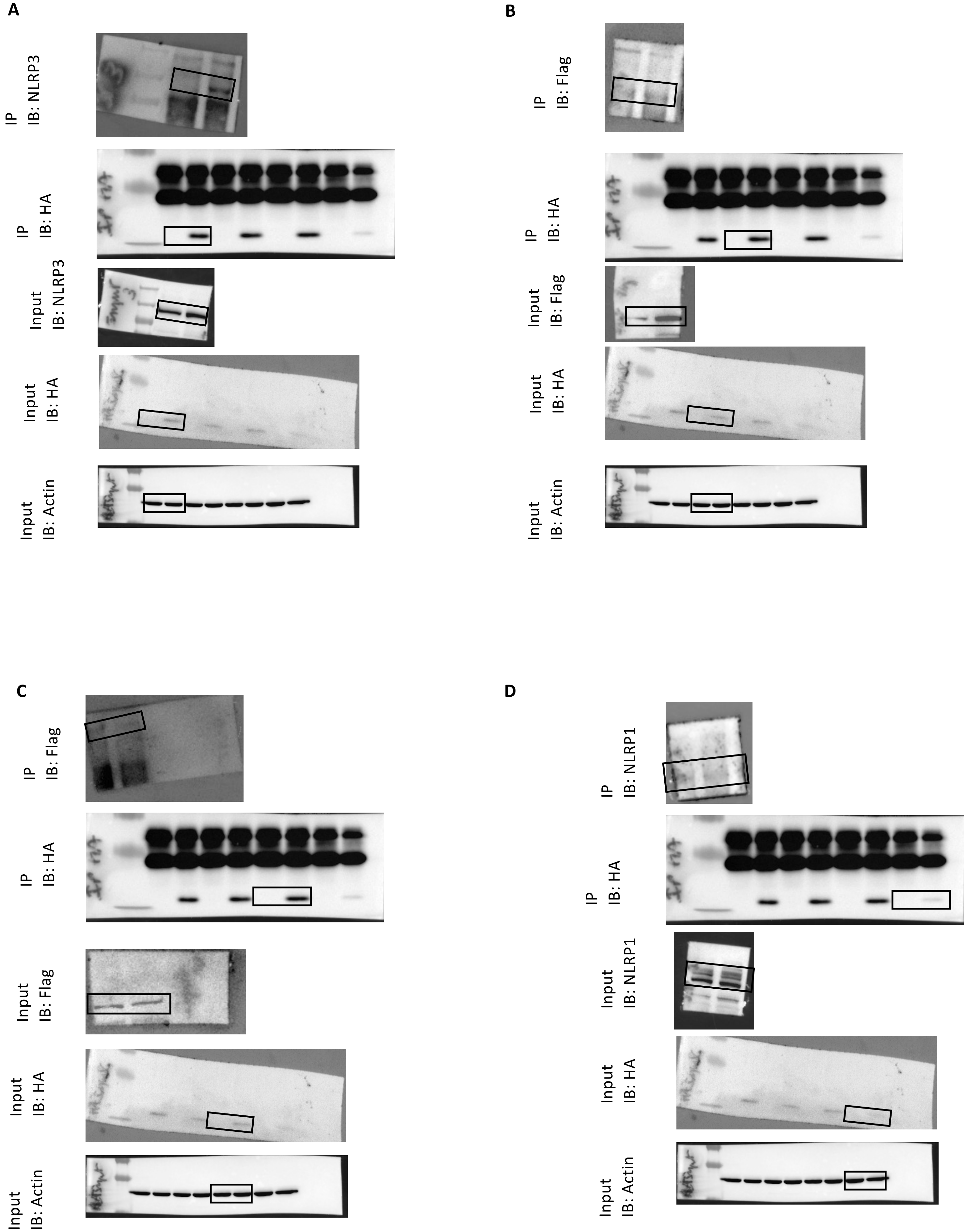

Supplement: Supplementary file 1 [file 41467_2026_78499_MOESM1_ESM.pdf]
