# Supplementary material for: Bacterial toxin sensing induces transient inflammasome-dependent innate immune memory that improves survival during sepsis
Source: Nat Commun. 2026 Oct 8;17:11460. doi: 10.1038/s41467-026-78499-5 (PMC13740286; doi:10.1038/s41467-026-78499-5)
Supplement: Supplementary file 2 [file 41467_2026_78499_MOESM2_ESM.pdf]

nature portfolio

Corresponding author(s): Laurent Boyer, Cédric Torre

Last updated by author(s): 07-01-2026

Reporting Summary

Nature Portfolio wishes to improve the reproducibility of the work that we publish. This form provides structure for consistency and transparency in reporting. For further information on Nature Portfolio policies, see our Editorial Policies and the Editorial Policy Checklist.

Please do not complete any field with "not applicable" or n/a. Refer to the help text for what text to use if an item is not relevant to your study.
For final submission: please carefully check your responses for accuracy; you will not be able to make changes later.

Statistics

For all statistical analyses, confirm that the following items are present in the figure legend, table legend, main text, or Methods section.

n/a	Confirmed	
☐	☑	The exact sample size (*n*) for each experimental group/condition, given as a discrete number and unit of measurement
☐	☑	A statement on whether measurements were taken from distinct samples or whether the same sample was measured repeatedly
☐	☑	The statistical test(s) used AND whether they are one- or two-sided<br>*Only common tests should be described solely by name; describe more complex techniques in the Methods section.*
☒	☐	A description of all covariates tested
☒	☐	A description of any assumptions or corrections, such as tests of normality and adjustment for multiple comparisons
☒	☐	A full description of the statistical parameters including central tendency (e.g. means) or other basic estimates (e.g. regression coefficient) AND variation (e.g. standard deviation) or associated estimates of uncertainty (e.g. confidence intervals)
☒	☐	For null hypothesis testing, the test statistic (e.g. *F*, *t*, *r*) with confidence intervals, effect sizes, degrees of freedom and *P* value noted<br>*Give P values as exact values whenever suitable.*
☒	☐	For Bayesian analysis, information on the choice of priors and Markov chain Monte Carlo settings
☒	☐	For hierarchical and complex designs, identification of the appropriate level for tests and full reporting of outcomes
☒	☐	Estimates of effect sizes (e.g. Cohen's *d*, Pearson's *r*), indicating how they were calculated

Our web collection on statistics for biologists contains articles on many of the points above.

Software and code

Policy information about availability of computer code

Data collection	
Data analysis	Data were analyzed using GraphPad Prism software v8.2.1 and Fiji v2.1.0 by ImageJ and FlowJo v10.6.2

For manuscripts utilizing custom algorithms or software that are central to the research but not yet described in published literature, software must be made available to editors and reviewers. We strongly encourage code deposition in a community repository (e.g. GitHub). See the Nature Portfolio guidelines for submitting code & software for further information.

Data

Policy information about availability of data

All manuscripts must include a data availability statement. This statement should provide the following information, where applicable:

- Accession codes, unique identifiers, or web links for publicly available datasets
- A description of any restrictions on data availability
- For clinical datasets or third party data, please ensure that the statement adheres to our policy

Reported in the section Materiel and Method, paragraph Data availability

All data supporting the findings of this study are available within the article and its supplementary information or from the corresponding author upon reasonable request. The ATACSeq data and RNASeq data generated in this study and shown in Figure 3 have been deposited in the GEO database. ATACSeq data are available using the following link https://www.ncbi.nlm.nih.gov/geo/query/acc.cgi?acc=GSE337610.
RNASeq data are available using the following link https://www.ncbi.nlm.nih.gov/geo/query/acc.cgi?acc=GSE337763.
Source data are provided with this paper.

Research involving human participants, their data, or biological material

Policy information about studies with human participants or human data. See also policy information about sex, gender (identity/presentation), and sexual orientation and race, ethnicity and racism.

Reporting on sex and gender	
Reporting on race, ethnicity, or other socially relevant groupings	
Population characteristics	
Recruitment	
Ethics oversight	

Note that full information on the approval of the study protocol must also be provided in the manuscript.

Field-specific reporting

Please select the one below that is the best fit for your research. If you are not sure, read the appropriate sections before making your selection.

☑ Life sciences ☐ Behavioural & social sciences ☐ Ecological, evolutionary & environmental sciences

For a reference copy of the document with all sections, see nature.com/documents/nr-reporting-summary-flat.pdf

Life sciences study design

All studies must disclose on these points even when the disclosure is negative.

Sample size	For experiments involving cells, n=3 was chosen as the minimal replicate number. For mice experiments, calculations were based on previous publication (Diabaté et al 2015; DOI: 10.1371/journal.ppat.1004732) and the G*Power software
Data exclusions	No data were excluded
Replication	Reported in the figures' legends. Experiements were repeted at least three times and performed independently
Randomization	Random allocation was not applicable to these experiments, as treatment and control conditions were performed in parallel using samples derived from the same donor, cell preparation, or experimental batch. To minimize potential sources of bias and technical variability, samples from each donor or experiment were distributed across the different experimental conditions and processed simultaneously using identical protocols and reagents. Where applicable, analyses were based on paired within-donor or within-experiment comparisons. Experimental conditions, treatment procedures, and analysis criteria were defined prospectively and applied consistently across all groups.
Blinding	Blinding was not feasible during the experimental procedures because investigators needed to know the treatment allocation to correctly administer the different conditioning treatments and perform the subsequent stimulation and sample-processing steps. To minimize potential bias, all experimental groups were processed in parallel according to standardized protocols, under matched experimental conditions and using predefined outcome measures. Whenever technically feasible, investigators were blinded to group allocation during data analysis.

Behavioural & social sciences study design

All studies must disclose on these points even when the disclosure is negative.

Study description	
Research sample	
Sampling strategy	
Data collection	
Timing	
Data exclusions	
Non-participation	
Randomization	

Ecological, evolutionary & environmental sciences study design

All studies must disclose on these points even when the disclosure is negative.

Study description	
Research sample	
Sampling strategy	
Data collection	
Timing and spatial scale	
Data exclusions	
Reproducibility	
Randomization	
Blinding	

Did the study involve field work? ☐ Yes ☐ No

Field work, collection and transport

Field conditions	
Location	
Access & import/export	
Disturbance	

Reporting for specific materials, systems and methods

We require information from authors about some types of materials, experimental systems and methods used in many studies. Here, indicate whether each material, system or method listed is relevant to your study. If you are not sure if a list item applies to your research, read the appropriate section before selecting a response.

Materials & experimental systems

n/a	Involved in the study	
☐	☑	Antibodies
☐	☑	Eukaryotic cell lines
☐	☐	Palaeontology and archaeology
☐	☑	Animals and other organisms
☐	☑	Clinical data
☐	☐	Dual use research of concern
☐	☐	Plants

Methods

n/a	Involved in the study	
☐	☐	ChIP-seq
☐	☑	Flow cytometry
☐	☐	MRI-based neuroimaging

Antibodies

Antibodies used	Reported in the section Material and Method, paragraphs ELISA and Immunoprecipitation
Validation	Antibodies were all validated by the manufacturer.

The following antibodies were used in this study: rabbit anti-IL-1β (GTX74034, Genetex at 1/1000), mouse anti-Rac (clone 102/Rac1, BD Biosciences at 1/1000), goat anti-Rac2 (ab2244, Abcam at 1/1000), mouse anti-NLRP3 (clone Cryo-2, Adipogen at 1/100 0), rabbit anti-Pak1 (2602, CST at 1/1000), rabbit anti-Pak2 (2608, CST at 1/1000), mouse anti-β-actin (AC-74, Sigma-Aldrich at 1/2000). anti-HA (901515, Biolegend8 at 1/1000) and anti-NLRP3 (AG-20B-0014-C100, Coger at 1/1000). anti-NLRP1 (679802, Biolegend at 1/1000). anti-β-actin (3700S, Cell Signalling technology at 1/1000).- anti-Flag (F1804, Sigma-Aldrich at 1/1000) and anti-BCL2A1 (kindly provided by Marco J. Herold, Melbourne, Australia at 1/100) anti-tubulin (T4026, Sigma at 1/1000).

Eukaryotic cell lines

Policy information about cell lines and Sex and Gender in Research

Cell line source(s)	HEK293T from ATCC CRL-3216, Immortalized BMDM kindly provided by Dr. Bénédicte P.<br>Reported in the section Material and Method, paragraph Cell model of trained immunity and Immunoprecipitation
Authentication	All cell lines were authenticated by using PCR with species specific primers
Mycoplasma contamination	Cells were tested negative for mycoplasma contamination
Commonly misidentified lines<br>(See ICLAC register)	No commonly misidentified cell lines were used

Palaeontology and Archaeology

Specimen provenance	
Specimen deposition	
Dating methods	

☐ Tick this box to confirm that the raw and calibrated dates are available in the paper or in Supplementary Information.

Ethics oversight	

Note that full information on the approval of the study protocol must also be provided in the manuscript.

Animals and other research organisms

Policy information about studies involving animals; ARRIVE guidelines recommended for reporting animal research, and Sex and Gender in Research

Laboratory animals	C57BL/6J mice ,C57BL/6J NLRP3 KO mice, C57BL/6J Pak1 KO mice, C57BL/6J GSDMD KO mice
Wild animals	Wild animals the study did not involved wild animals
Reporting on sex	This study involves 6-week-old female C57BL/6J mice (Janvier labs)
Field-collected samples	The study did not involved sample collected from the field
Ethics oversight	Reported in the section Material and Method, paragraph Ethics statement. This study was carried out in strict accordance with the guidelines of the Council of the European Union (Directive 86/609/EEC) regarding the protection of animals used for experimental and other scientific purposes. The protocol was approved by the Institutional Animal Care and Use Committee on the Ethics of Animal Experiments of Nice, France (APAFIS#18322-2018121809942703 5 v2 and APAFIS#24906-2020031614223228 v2). The study was conducted in accordance with French legislation and the principles of the Declaration of Helsinki with the informed consent obtained from the blood donors. Human blood from healthy donors was obtained from the Etablissement Français du Sang (N13002).

Note that full information on the approval of the study protocol must also be provided in the manuscript.

Clinical data

Policy information about clinical studies

All manuscripts should comply with the ICMJE guidelines for publication of clinical research and a completed CONSORT checklist must be included with all submissions.

Clinical trial registration	Human blood from healthy donors was obtained from the Etablissement Français du Sang (N13002)
Study protocol	Buffy coats from volunteers were ordered from the French blood establishment. PBMC were isolated using Ficoll (Cytiva), and red cells were lysed using lysing buffer (BD Biosciences). Then, monocytes were purified using anti-CD14 microbeads (Miltenyi) and autoMACS Pro Separator (Miltenyi) according to the manufacturer's instructions. Monocytes were cultivated in RPMI 1640 GlutaMax medium (Life Technologies) supplemented with 10 % (vol/vol) heat-inactivated FBS (Biowest) and 50 µg/mL gentamycin (Life Technologies) at 37°C in an atmosphere containing 5 % CO2. Macrophage differentiation was induced by addition of CSF-1 (100 ng/mL, Miltenyi) into the culture medium.
Data collection	Genes expression were analyzed using qPCR for Il-1b, Il-6 and Tnf-a
Outcomes	We observed a CNF1-triggered trained phenotype with an increased IL-1B, IL-6 and TNF-a expression in human monocyte-derived macrophages (hMDM) that were significantly reduced in Bcl2a1 knockdown condition.

Dual use research of concern

Policy information about dual use research of concern

Hazards

Could the accidental, deliberate or reckless misuse of agents or technologies generated in the work, or the application of information presented in the manuscript, pose a threat to:

No | Yes

- [ ] [ ] Public health
- [ ] [ ] National security
- [ ] [ ] Crops and/or livestock
- [ ] [ ] Ecosystems
- [ ] [ ] Any other significant area

Experiments of concern

Does the work involve any of these experiments of concern:

No | Yes

- [ ] [ ] Demonstrate how to render a vaccine ineffective
- [ ] [ ] Confer resistance to therapeutically useful antibiotics or antiviral agents
- [ ] [ ] Enhance the virulence of a pathogen or render a nonpathogen virulent
- [ ] [ ] Increase transmissibility of a pathogen
- [ ] [ ] Alter the host range of a pathogen
- [ ] [ ] Enable evasion of diagnostic/detection modalities
- [ ] [ ] Enable the weaponization of a biological agent or toxin
- [ ] [ ] Any other potentially harmful combination of experiments and agents

Plants

Seed stocks

Novel plant genotypes

Authentication

ChIP-seq

Data deposition

- [ ] Confirm that both raw and final processed data have been deposited in a public database such as GEO.
- [ ] Confirm that you have deposited or provided access to graph files (e.g. BED files) for the called peaks.

Data access links
May remain private before publication.

Files in database submission

Genome browser session
(e.g. UCSC)

Methodology

Replicates

Sequencing depth

Antibodies

Peak calling parameters

Data quality

Software

Flow Cytometry

Plots

Confirm that:

- [x] The axis labels state the marker and fluorochrome used (e.g. CD4-FITC).
- [x] The axis scales are clearly visible. Include numbers along axes only for bottom left plot of group (a 'group' is an analysis of identical markers).
- [x] All plots are contour plots with outliers or pseudocolor plots.
- [x] A numerical value for number of cells or percentage (with statistics) is provided.

Methodology

Sample preparation: Reported in the section Material and Method, paragraph Flow cytometry analysis

Instrument: Flow cytometry was performed using an Aurora flow cytometer from Cytek.

Software: Flow cytometry data were analyzed using FlowJo v10.6.2

Cell population abundance: For each sample, at least 10 000 CD45+ expressing cells were analysed

Gating strategy: Reported in supplemental figure 4

- [x] Tick this box to confirm that a figure exemplifying the gating strategy is provided in the Supplementary Information.

Magnetic resonance imaging

Experimental design

Design type

Design specifications

Behavioral performance measures

Imaging type(s)

Field strength

Sequence & imaging parameters

Area of acquisition

Diffusion MRI ☐ Used ☐ Not used

Preprocessing

Preprocessing software

Normalization

Normalization template

Noise and artifact removal

Volume censoring

Statistical modeling & inference

Model type and settings

Effect(s) tested

Specify type of analysis: ☐ Whole brain ☐ ROI-based ☐ Both

Statistic type for inference

(See Eklund et al. 2016)

Correction

Models & analysis

n/a	Involved in the study
☐	☐ Functional and/or effective connectivity
☐	☐ Graph analysis
☐	☐ Multivariate modeling or predictive analysis

Functional and/or effective connectivity

Graph analysis

Multivariate modeling and predictive analysis

This checklist template is licensed under a Creative Commons Attribution 4.0 International License, which permits use, sharing, adaptation, distribution and reproduction in any medium or format, as long as you give appropriate credit to the original author(s) and the source, provide a link to the Creative Commons license, and indicate if changes were made. The images or other third party material in this article are included in the article's Creative Commons license, unless indicated otherwise in a credit line to the material. If material is not included in the article's Creative Commons license and your intended use is not permitted by statutory regulation or exceeds the permitted use, you will need to obtain permission directly from the copyright holder. To view a copy of this license, visit http://creativecommons.org/licenses/by/4.0/
