# Supplementary material for: Bacterial toxin sensing induces transient inflammasome-dependent innate immune memory that improves survival during sepsis
Source: Nat Commun. 2026 Oct 8;17:11460. doi: 10.1038/s41467-026-78499-5 (PMC13740286; doi:10.1038/s41467-026-78499-5)
Supplement: Supplementary file 3 [file 41467_2026_78499_MOESM3_ESM.pdf]

nature portfolio

Peer Review File

Bacterial toxin sensing induces transient inflammasome-dependent innate immune memory that improves survival during sepsis

Corresponding Author: Dr Laurent Boyer

This file contains all reviewer reports in order by version, followed by all author rebuttals in order by version.

Version 0:

Reviewer comments:

Reviewer #1

(Remarks to the Author)
The authors demonstrate that CNF1 toxin induces trained immunity in macrophages from various sources and further establish that this induction is dependent on the NLRP3 inflammasome and IL-1 signaling pathway. However, both Figure 1 and Figure 2 rely exclusively on qPCR for analysis, with a notable absence of ELISA data. In Figure 3, the authors employ ATAC-seq and RNA-seq to identify Bcl2a1 as a key regulator of trained immunity through siRNA screening. Figure 4 confirms that Bcl2a1 is essential for trained immunity and interacts with NLRP3. Finally, Figure 5 validates that CNF1-induced trained immunity alters monocyte proportions and improves survival rates in sepsis. The following suggestions may help the authors enhance the rigor of the manuscript.

Q1: Line 95, the toxin is intravenously injected at 250 ng/kg, followed by the isolation of splenic macrophages. Could you explain why splenic macrophages were chosen instead of other types of macrophages?

Q2: Figures 1 and 2 present only qPCR results, with a lack of ELISA data. qPCR is primarily used to detect mRNA levels of genes, and gene expression is often subject to complex regulation, which may not directly reflect protein expression. In contrast, ELISA provides accurate data for both quantitative and qualitative analysis of proteins.

Q3: In Figure 3F, the IL6 expression heatmap lacks a corresponding legend, making it difficult to interpret the relationship between color and IL6 concentration. Additionally, the poor repeatability in the three experimental replicates may lead to imprecise results. In fact, during the siRNA screening, more than half of the genes, including Bcl2a1b, Ass1, Nes, Akap6, and Macf1, exhibited a decrease in IL6 concentration post-siRNA treatment. The potential interactions or regulatory effects among these genes were not addressed? Why do the authors consider Bcl2a1 to be a key regulator of CNF1-induced trained immunity?

Q4: As shown in Figure 4H, Bcl2a1 interacts with NLRP3. Is it possible that other genes such as Ass1, Nes, Akap6, Macf1, and potentially more could also interact with NLRP3?

Q5: In Figure 5, “WT C57BL/6J mice were trained with intravenous injection of CNF1 or its catalytic inactive mutant (CNF1 C866S) (250 ng/kg) for 14 days before bone marrow cells were isolated and analyzed.” It is recommended to include different time points, as this could provide a more precise understanding of the dynamic changes in immune cell proportions.

Q6: Studies have shown that low concentrations of LPS can also induce trained immunity. In the sepsis model, the authors could consider comparing low concentrations of LPS with CNF1 toxin, while also including β-glucan as a positive control for trained immunity. This would allow for the analysis of the protective effects across different groups and provide a foundation for practical applications.

Reviewer #2

(Remarks to the Author)
In this manuscript, Torre et al. investigate if activation of the NLRP3 inflammasome by E. coli CNF1 toxin results in trained

immunity. Evidence was presented that CNF1 imparted trained immunity on murine macrophages in vivo and in vitro, as revealed by increased cytokine (IL-1b, IL-6, TNFa) gene expression in response to LPS. The effect appears to require NLRP3 inflammasome activation, IL-1b release and IL1R signaling in vitro. RNAseq and ATAseq were used to identify genes impacted by trained immunity. An siRNA screen performed on the gene candidates identified the anti-apoptotic protein Bcl2A1 as important for trained immunity in murine macrophages. Results with human macrophages confirmed a similar outcome. Overexpressed NLRP3 and Bcl2A1 were shown to interact in HEK293T cells. However, it is unclear if Bcl2A1 and NLRP3 interact in macrophages or if Bcl2A1 regulates NLRP3 in macrophages. Finally, CNF1 treatment of mice resulted in increased myeloid progenitors cells and monocyte in bone marrow and increased resistance to lethal systemic infection with E. coli.

Overall, the results are interesting and potentially an important demonstration that effector-triggered responses can result in trained immunity. The manuscript needs to be edited for clarity and to resolve errors. Data showing the importance of Bcl2A1 for NLRP3 inflammasome activation needs to included.

Major points:
Fig.1 and all other bar graphs do not show the individual data points used to calculate the means +/- SEM, which is now considered standard for scientific rigor. In addition, legends should specifically state how many independent experiments were performed to generate the data points.
Lines 98-99: Supp Fig.1A is a good opportunity to demonstrate that the purified CNF1 and mutant C866S proteins are of similar purity, solubility and stability. It would make sense to introduce BMDMs here.
Lines 99-101: Supp Fig.1B shows results of in vitro BMDM infections not in vivo infections.
It is not clear why the data shown in Supp Fig.1 C-H is not discussed.
Add statistical analysis to Fig.2A-O.
Line 140: Supp Fig.2 does not show the nigericin experiments (Bcl2A1 data is shown). The nigericin data is in Fig.2 S-U.
Supp Fig.2: The title is not correct, since inflammasome activation was not measured. The authors need to
Lines 172-180, Fig.4C,F: The text should state that there was a trend with the tnfa data but it was not significant.
Line 182: Introduce the function of Bcl2A1.
Line 183: Mention that these are HEK293T experiments and can detect low affinity interactions. If experiments with macrophages were carried out to detect interaction of NLRP3 and Bcl2A1 and were unsuccessful this should also be stated.
Line 196: Define CMP.
Fig.5N: The legend should indicate if the data is from one experiment, and if the experiment was repeated for reproducibility.

Minor points:
Line 102: The sentence “We then made the parallel…” needs rephrasing.
Line 107: Add that treatment was done with different amounts of the toxins.
Line 115: No significant increase in IL-6 expression is seen in Fig.1M.
Line 123: Mention BMDMs were used in Fig.2.
Describe how statistical significance was determined and presented for Fig.3E.
Line 187: change “lacking” to “with”.
Fig.4H: The vertical IP bracket line should extend down to the IB:HA panel.
Line 197: Add reference for the previous works.

Reviewer #3

(Remarks to the Author)
Summary of the work and overall evaluation:

Memory in the adaptive immune system against bacterial toxins is well established. This study demonstrates that the innate immune system can also undergo epigenetic training following exposure to a bacterial toxin, using the Escherichia coli toxin CNF1 as a model.

Macrophages exposed to CNF1 develop a trained phenotype and mount a stronger inflammatory response upon subsequent bacterial challenge, even when the bacteria lack CNF1. This enhanced response protects animals in a model of E. coli-triggered sepsis.

Mechanistically, toxin-induced training is mediated through inflammasome activation and IL-1-dependent epigenetic reprogramming of innate progenitors. While this pathway is consistent with previously described mechanisms of innate immune training in other inflammatory contexts, its application to bacterial toxin sensing and sepsis represents a novel and important contribution.
The work convincingly shows that bacterial toxin detection and effector-triggered immunity can induce innate immune training. The following questions could further strengthen the study:

1. Universality of inflammasome-induced training
Is innate training a general consequence of inflammasome activation and IL-1 secretion? For example, can it be induced by other inflammasomes activated by bacterial signals (e.g., flagellin, dsDNA) or by sterile NLRP3 activators such as ATP? Is training enhanced by co-detection of alarmins released during lytic cell death?

2. IL-1β versus IL-18 signalling
Recombinant IL-1β alone appears sufficient to induce monocyte training, whereas IL-18 does not, despite overlapping downstream signalling pathways. Do monocytes/macrophages in these cultures express IL-18 receptor?

3. Specificity of the training signature
ATAC-seq identifies genes induced by toxin training. Is this transcriptional/epigenetic signature shared with other training models (e.g., BCG or β-glucan), or is it stimulus-specific?

4. Role of Bcl2a1 in training
Toxin-triggered training is partially dependent on Bcl2a1. Is Bcl2a1 also required for BCG- or β-glucan–induced training, or is its function specific to toxin-mediated training?

5. Interaction with NLRP3
The potential interaction between Bcl2a1 and NLRP3 is intriguing. Does Bcl2a1 interact with the NACHT domain of other NLR family members, or is this interaction unique to NLRP3?

6. Duration and anatomical site of memory
How long does toxin-induced memory persist in vivo? Does training occur primarily in bone marrow progenitors or in tissue-resident macrophages, i.e. does memory persist after transplantation of trained bone marrow into naïve recipients?

7. Timing of the training window
Is there a defined temporal window during which IL-1 signalling induces training in vivo after toxin detection? For example, in stroke models, IL-1 trains bone marrow progenitors only within the first week; blockade during that window prevents training, whereas later inhibition of IL-1 does not (PMID: 39043180).

8. Risk of immunopathology
Could toxin-induced training predispose developing monocytes to exaggerated responses to non-infectious triggers (e.g., MSU crystals in vivo)? If so, this might increase susceptibility to inflammatory comorbidities, similar to what was observed after stroke-induced innate training. Are there any data on this in the human population that could be analysed (I understand this may not be available)?

Version 1:

Reviewer comments:

Reviewer #1

(Remarks to the Author)
All previous points have been adequately responded to. No more comments.

Reviewer #2

(Remarks to the Author)
The authors have addressed all comments except one and significantly improved the manuscript.

The only comment that was not addressed was in the Overall section and asked for "Data showing the importance of Bcl2A1 for NLRP3 inflammasome activation". Unless I am missing something I could not find this experimental result in the revised manuscript. Unless the authors can argue otherwise I think this is an important experiment and straightforward since Bcl2a1-/- macrophages are available.

Reviewer #3

(Remarks to the Author)
I thank the authors for their thorough and thoughtful revision. The revised manuscript is substantially improved and addresses the vast majority of the concerns raised during the first round of review.

I have one remaining conceptual concern regarding the interpretation of the findings that I believe should be addressed through important revisions to the text before publication.

Major comment

The revised manuscript convincingly demonstrates induction of an innate trained phenotype following CNF1 exposure. The combination of a resting phase between stimuli, enhanced secondary responses, epigenetic remodelling (ATAC-seq), transcriptional reprogramming, and IL-1/NLRP3 dependence is consistent with current definitions of trained immunity.

However, I do not believe the current data support conclusions implying durable central trained immunity or long-lived bone marrow-resident immune memory, as described for classical models such as BCG, β-glucan or Western diet-induced

training.

The additional experiments introduced during revision show that:

1. the trained phenotype is detectable at 1–2 weeks but is no longer statistically significant at 3–4 weeks;
2. alterations in myeloid cell composition are similarly transient;
3. bone marrow transplantation experiments, which require 4 weeks to engraft, were unable to transfer the phenotype to naïve recipients.

Taken together, these observations suggest that CNF1 induces a transient trained immune programme rather than durable hematopoietic reprogramming comparable to that described following BCG or β-glucan exposure.

While bone marrow reprogramming is not a formal requirement for defining trained immunity, it has become a hallmark of persistent central in vivo trained immunity. The current study therefore does not establish that the long-term memory resides within long-lived hematopoietic progenitors.

Accordingly, I recommend that the authors moderate several statements throughout the manuscript.

Specific suggestions:

1. The current title refers to "innate immune memory," which is acceptable. However, I recommend avoiding wording that may imply durable or long-lived memory. An alternative such as "Bacterial toxin sensing induces transient inflammasome-dependent trained immunity that improves survival during sepsis" may better reflect the data.

2. I recommend modifying the final sentence of the Abstract to acknowledge the transient nature of the response. For example: These findings identify toxin-driven inflammasome activation as a trigger of transient trained immunity and demonstrate that this response enhances resistance during experimental bacterial sepsis.

3. The Results section entitled "CNF1 toxin induces central TI and protects against lethal infection" appears stronger than warranted. A title such as "CNF1 induces transient myeloid reprogramming and protects against lethal infection" would be more consistent with the data. Likewise, the paragraph discussing the increase in CMPs should be softened. Statements suggesting that long-lived hematopoietic stem cells explain the persistence of immune memory are speculative and are not supported by the presented data, particularly in light of the transient phenotype and the inability of bone marrow transplantation to transfer the phenotype.

4. The Discussion would benefit from explicitly acknowledging that the trained phenotype is reversible. Rather than presenting this as a limitation, I think this could become one of the conceptual strengths of the study. The authors could discuss the possibility that bacterial toxins induce a transient, self-limited form of trained immunity that provides short-term protection against recurrent infection while minimising the risks associated with prolonged inflammatory reprogramming. This interpretation would also fit well with the newly added discussion regarding potential immunopathology.

Version 2:

Reviewer comments:

Reviewer #2

(Remarks to the Author)
The authors addressed the remaining concern of determining the importance of Bcl2A1 for NLRP3 inflammasome activation.

Open Access This Peer Review File is licensed under a Creative Commons Attribution 4.0 International License, which permits use, sharing, adaptation, distribution and reproduction in any medium or format, as long as you give appropriate credit to the original author(s) and the source, provide a link to the Creative Commons license, and indicate if changes were made.
In cases where reviewers are anonymous, credit should be given to 'Anonymous Referee' and the source.
The images or other third party material in this Peer Review File are included in the article's Creative Commons license, unless indicated otherwise in a credit line to the material. If material is not included in the article's Creative Commons license and your intended use is not permitted by statutory regulation or exceeds the permitted use, you will need to obtain permission directly from the copyright holder.
To view a copy of this license, visit https://creativecommons.org/licenses/by/4.0/

Please find below our point-by-point response to the reviewer's comments. We thank the reviewer for their time and constructive comments, which have helped us improve our work.

REVIEWER COMMENTS

Reviewer #1 (Remarks to the Author):

The authors demonstrate that CNF1 toxin induces trained immunity in macrophages from various sources and further establish that this induction is dependent on the NLRP3 inflammasome and IL-1 signaling pathway. However, both Figure 1 and Figure 2 rely exclusively on qPCR for analysis, with a notable absence of ELISA data. In Figure 3, the authors employ ATAC-seq and RNA-seq to identify Bcl2a1 as a key regulator of trained immunity through siRNA screening. Figure 4 confirms that Bcl2a1 is essential for trained immunity and interacts with NLRP3. Finally, Figure 5 validates that CNF1-induced trained immunity alters monocyte proportions and improves survival rates in sepsis. The following suggestions may help the authors enhance the rigor of the manuscript.

Q1: Line 95, the toxin is intravenously injected at 250 ng/kg, followed by the isolation of splenic macrophages. Could you explain why splenic macrophages were chosen instead of other types of macrophages?

We thank the reviewer for this question which allowed us to clarify the rationale underlying our experimental model. Mice were intravenously injected with either wild-type CNF1 toxin or its catalytic inactive mutant. We then isolated splenic macrophages to perform ex vivo experiments due to their strong physiological relevance in blood-borne infections (Borges da Silva H et al., 2015). Splenic macrophages play a key role in the detection and clearance of circulating pathogens and in initiating appropriate adaptive immune responses (Oriana A. Perez et al., 2017). In addition, splenic macrophages are easily isolated from the spleen, providing a high cellular yield from a single animal, therefore improving experimental reproducibility. We have now included this information in the text (page 5 line 96-98).

Q2: Figures 1 and 2 present only qPCR results, with a lack of ELISA data. qPCR is primarily used to detect mRNA levels of genes, and gene expression is often subject to complex regulation, which may not directly reflect protein expression. In contrast, ELISA provides accurate data for both quantitative and qualitative analysis of proteins.

We fully agree with the reviewer that ELISA measurements provide a more direct assessment of cytokine production at the protein level. However, trained immunity is primarily driven by epigenetic and transcriptional reprogramming, and measurement of cytokine mRNA expression by qPCR after restimulation is widely used as a standard readout in the field. Nevertheless, to directly address the reviewer's concern, we have now added ELISA data in (Figure 1 L), demonstrating increased cytokine secretion following CNF1-mediated training. These results are fully consistent with the transcriptional changes observed by qPCR and further strengthen our conclusions (page 5 line 122-124).

Q3: In Figure 3F, the IL6 expression heatmap lacks a corresponding legend, making it difficult to interpret the relationship between color and IL6 concentration. Additionally, the poor repeatability in the three experimental replicates may lead to imprecise results. In fact, during the siRNA screening, more than half of the genes, including Bcl2a1b, Ass1, Nes, Akap6, and Macf1, exhibited a decrease in IL6 concentration post-siRNA treatment. The potential interactions or regulatory effects among these genes were not addressed? Why do the authors consider Bcl2a1 to be a key regulator of CNF1-induced trained immunity?

We agree with the reviewer, and we have now improved Figure 3 by adding a color scale to the heatmap, with green indicating higher fold changes and red indicating lower fold changes, thereby facilitating the interpretation of IL-6 levels.
We agree that several genes identified in the siRNA screen, including Ass1, Nes, Akap6, Macf1, and Bcl2a1b, reduced IL-6 production upon silencing, suggesting that multiple pathways may contribute to the trained immunity phenotype. Among these candidates, Bcl2a1 emerged as the most robust hit. We focused on Bcl2a1 since all three independent siRNAs targeting Bcl2a1 consistently produced the strongest reduction in IL-6 production (Figure 3E), a hallmark of trained immunity. Importantly, RNA-seq analysis revealed that several functional Bcl2a1 members (Bcl2a1a, Bcl2a1b, and Bcl2a1d) were induced following CNF1-mediated training, suggesting coordinated regulation of the Bcl2a1 locus. Finally, previous studies have established that these homologous genes encode functional pro-survival proteins with overlapping activities (Schenk et al., 2017), supporting the biological relevance of this pathway. Together, these observations led us to prioritize Bcl2a1 as a key regulator of CNF1-induced trained immunity.
In this revised version we have clarified this rationale (page 7, lines 183–191).

Q4: As shown in Figure 4H, Bcl2a1 interacts with NLRP3. Is it possible that other genes such as Ass1, Nes, Akap6, Macf1, and potentially more could also interact with NLRP3?

We thank the reviewer for this interesting suggestion. To address this question, we investigated whether the other candidate genes identified in our screen, namely Ass1, Nes, Akap6, and Macf1, could also interact with NLRP3. To this end, we performed co-immunoprecipitation experiments in both HEK293T cells (A-D) and immortalized bone marrow-derived macrophages (iBMDMs) stably expressing Flag-NLRP3 (E-I). We were unable to detect a convincing interaction between NLRP3 with AKAP6 (A-E), MACF1 (C-G) nor Nestin (D-H) in both cell types. In contrast, we could observe a co-immunoprecipitation between NLRP3 and ASS1 in iBMDM cells (F).
A link functional link between ASS1 and NLRP3 has been previously proposed in the context of atherosclerotic inflammation (WU, Front. Biosci. (Landmark Ed) 2026; 31(3): 47686) and further study would be required to determine its role during trained immunity. Altogether, these results suggest that in addition to Bcl2a1, Ass1 might be involved in NLRP3 inflammasome signaling complex whereas Akap6, Nestin and Macf1 are unlikely to participate directly in the same signaling complex. Nevertheless, we cannot exclude the possibility that these genes regulate trained immunity through different mechanisms or indirectly modulate inflammasome activation.

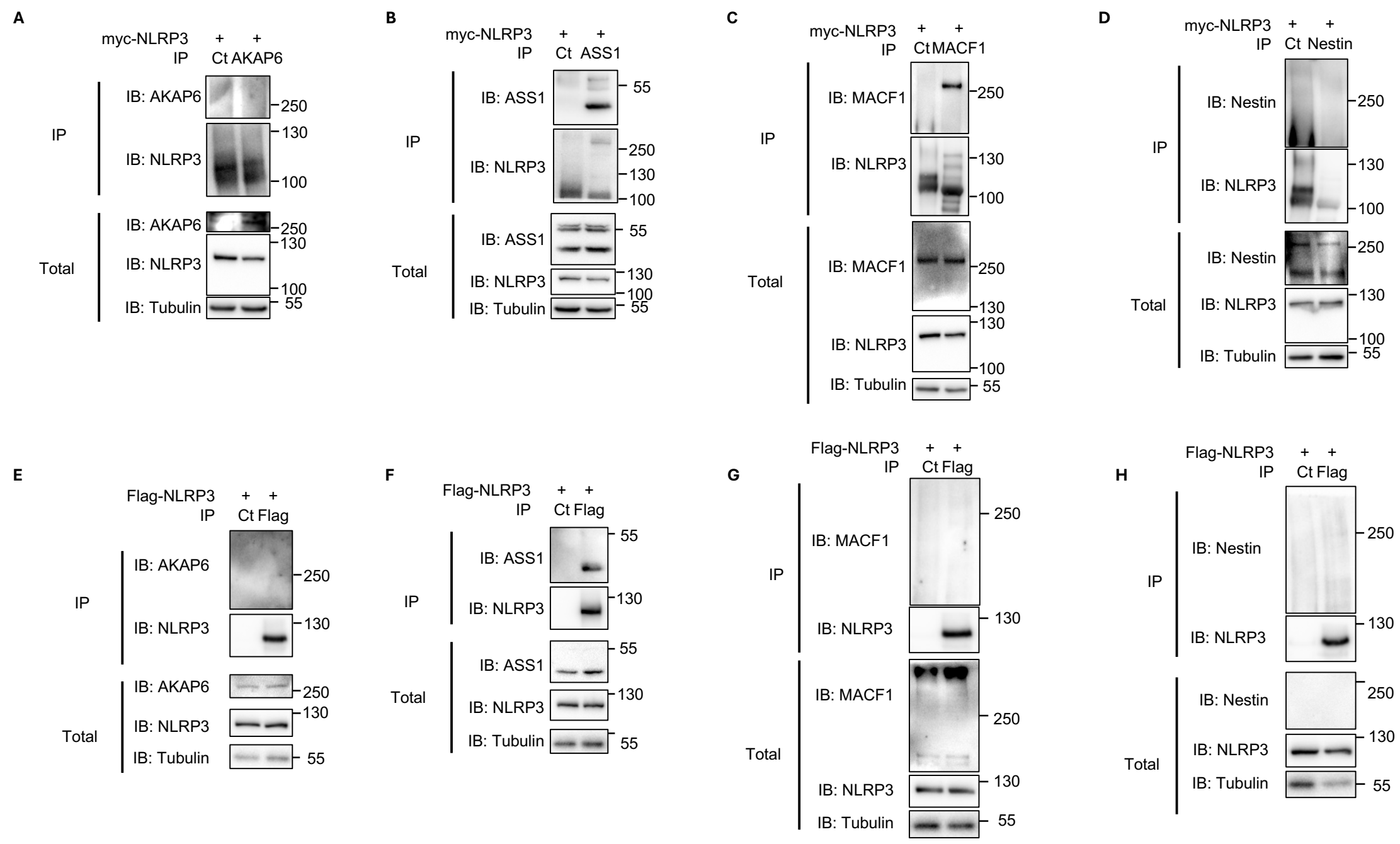

Other interactants of NLRP3 inflammasome

(A - D) HEK293T cells were transfected with plasmids encoding Myc-NLRP3. Cell lysates were processed for immunoprecipitation using IgG control (Ct) as negative control or anti-AKAP6 antibody (A), anti-ASS1 antibody (B), anti-MACF1 antibody (C), or anti-Nestin antibody (D). Experiments were repeated at least three times. (E-I) Flag-NLRP3 expressing iBMDM cells were lysed and processed for immunoprecipitation (IP) using control (Ct) or Flag-beads. Experiments were repeated at least three times.

Q5: In Figure 5, “WT C57BL/6J mice were trained with intravenous injection of CNF1 or its catalytic inactive mutant (CNF1 C866S) (250 ng/kg) for 14 days before bone marrow cells were isolated and analyzed.” It is recommended to include different time points, as this could provide a more precise understanding of the dynamic changes in immune cell proportions.

We thank the reviewer for this valuable suggestion. Following the reviewer’s advice and to better characterize the kinetics of the trained immunity phenotype induced by CNF1, we extended our analysis to additional time points up to 4 weeks. In addition to the 14-day time point shown in initial in Figure 5, we analyzed cytokine responses and observed similar statistically significant changes at 1 and 2 weeks after training. In contrast, these changes were no longer statistically significant at 3 and 4 weeks post-training, indicating that the cytokine component of CNF1-induced trained immunity is transient. Interestingly, changes in circulating immune cell populations are delayed. Alterations in blood cell proportions became apparent at 2 and 3 weeks after training but were no longer detectable at 4 weeks. These findings suggest that functional reprogramming of innate immune responses precedes the

remodeling of circulating immune cell populations and that both processes are reversible over time. We have now included these additional data in (Supplemental Figure 5) and clarified the temporal evolution of the phenotype in the revised manuscript (page 9, lines 249–259).

Q6: Studies have shown that low concentrations of LPS can also induce trained immunity. In the sepsis model, the authors could consider comparing low concentrations of LPS with CNF1 toxin, while also including β-glucan as a positive control for trained immunity. This would allow for the analysis of the protective effects across different groups and provide a foundation for practical applications.

We thank the reviewer for this insightful suggestion. We agree that a direct *in vivo* comparison of CNF1 with established trained immunity stimuli such as β-glucan and low-dose LPS would provide valuable insight into the relative efficacy and underlying mechanisms of these distinct training stimuli. However, establishing a meaningful *in vivo* comparison would require extensive optimization of dosing regimens and kinetics for each stimulus, followed by rigorous side-by-side evaluation in our sepsis model. Such a study would constitute a substantial research project in its own and would also require additional animal ethics approvals. Consequently, we believe that these experiments fall beyond the scope and timeframe of the current revision.

Reviewer #2 (Remarks to the Author):

In this manuscript, Torre et al. investigate if activation of the NLRP3 inflammasome by E. coli CNF1 toxin results in trained immunity. Evidence was presented that CNF1 imparted trained immunity on murine macrophages in vivo and in vitro, as revealed by increased cytokine (IL-1b, IL-6, TNFa) gene expression in response to LPS. The effect appears to require NLRP3 inflammasome activation, IL-1b release and IL1R signaling in vitro. RNAseq and ATAseq were used to identify genes impacted by trained immunity. An siRNA screen performed on the gene candidates identified the anti-apoptotic protein Bcl2A1 as important for trained immunity in murine macrophages. Results with human macrophages confirmed a similar outcome. Overexpressed NLRP3 and Bcl2A1 were shown to interact in HEK293T cells. However, it is unclear if Bcl2A1 and NLRP3 interact in macrophages or if Bcl2A1 regulates NLRP3 in macrophages. Finally, CNF1 treatment of mice resulted in increased myeloid progenitors cells and monocyte in bone marrow and increased resistance to lethal systemic infection with E. coli.

Overall, the results are interesting and potentially an important demonstration that effector-triggered responses can result in trained immunity. The manuscript needs to be edited for clarity and to resolve errors. Data showing the importance of Bcl2A1 for NLRP3 inflammasome activation needs to included.

Major points:

Fig.1 and all other bar graphs do not show the individual data points used to calculate the means +/- SEM, which is now considered standard for scientific rigor. In addition, legends should specifically state how many independent experiments were performed to generate the data points.
Lines 98-99: Supp Fig.1A is a good opportunity to demonstrate that the purified CNF1 and mutant C866S proteins are of similar purity, solubility and stability. It would make sense to introduce BMDMs here
Lines 99-101: Supp Fig.1B shows results of in vitro BMDM infections not in vivo infections.
It is not clear why the data shown in Supp Fig.1 C-H is not discussed.
We thank the reviewer for this very useful comment. The changes have been made accordingly along the text and figures as requested. Individual data points have now been added to all bar graphs throughout the manuscript, and figure legends have been revised to clearly indicate the number of independent experiments used to generate each dataset. As suggested, Supplementary Figure 1A has been expanded to better document the quality of the purified CNF1 and C866S proteins. We now provide evidence that both proteins display comparable purity, solubility and stability at different storage temperatures. BMDMs are also introduced at this stage to improve the flow of the Results section. We thank the reviewer for pointing out the inconsistency in lines 99–101. The text has been corrected to accurately indicate that the new Supplementary Figure 1D corresponds to *in vitro* BMDM infections. Finally, we apologize for the data presented in Supplementary Figure 1C–H that were redundant to main figure 1 and removed in the new Supplementary Figure 1.
The new results are presented in the novel Supplementary Figure 1 and discussed in the revised manuscript to provide additional context and improve clarity (p5 Line 103-112).

Add statistical analysis to Fig.2A-O.
The statistics have been added in the revised version.

Line 140: Supp Fig.2 does not show the nigericin experiments (Bcl2A1 data is shown). The nigericin data is in Fig.2 S-U.

The text has been corrected in the revised version

Supp Fig.2: The title is not correct, since inflammasome activation was not measured. The authors need to
The title has been changed in the revised version

Lines 172-180, Fig.4C,F: The text should state that there was a trend with the tnfa data but it was not significant.
The text has been corrected in the revised version

Line 182: Introduce the function of Bcl2A1.
The function of BCL2A1 has been added in the text in the revised version (p7, line 187-191 and p8 line 209-211).

Line 183: Mention that these are HEK293T experiments and can detect low affinity interactions. If experiments with macrophages were carried out to detect interaction of NLRP3 and Bcl2A1 and were unsuccessful this should also be stated.
We thank the reviewer for this useful comment. We have performed co-IP experiment using macrophages iBMDM stably expressing FLAG-NLRP3 and found a specific interaction with endogenous BCL2A1. The data has now been included as a new Figure 4G. In addition, we have revised the text introducing the Co-IP assay performed in HEK293T to highlight that this technique is well suited for detecting low-affinity interactions: "Using an overexpression system in HEK293T cells to facilitate the detection of weak interactions". (p8, line 213-221)

Line 196: Define CMP. The text has been revised to define CMP (p8, line 237-240)

Fig.5N: The legend should indicate if the data is from one experiment, and if the experiment was repeated for reproducibility. The text has been modified accordingly in the revised version

Minor points:

Line 102: The sentence "We then made the parallel…" needs rephrasing.
Done

Line 107: Add that treatment was done with different amounts of the toxins.
Done

Line 115: No significant increase in IL-6 expression is seen in Fig.1M.
Done

Line 123: Mention BMDMs were used in Fig.2.
Done

Describe how statistical significance was determined and presented for Fig.3E.
Done

Line 187: change "lacking" to "with".
Done

Fig.4H: The vertical IP bracket line should extend down to the IB:HA panel.
Done

Line 197: Add reference for the previous works.
Done

Reviewer #3 (Remarks to the Author):

Summary of the work and overall evaluation:

Memory in the adaptive immune system against bacterial toxins is well established. This study demonstrates that the innate immune system can also undergo epigenetic training following exposure to a bacterial toxin, using the Escherichia coli toxin CNF1 as a model.

Macrophages exposed to CNF1 develop a trained phenotype and mount a stronger inflammatory response upon

subsequent bacterial challenge, even when the bacteria lack CNF1. This enhanced response protects animals in a model of E. coli-triggered sepsis.

Mechanistically, toxin-induced training is mediated through inflammasome activation and IL-1-dependent epigenetic reprogramming of innate progenitors. While this pathway is consistent with previously described mechanisms of innate immune training in other inflammatory contexts, its application to bacterial toxin sensing and sepsis represents a novel and important contribution.

The work convincingly shows that bacterial toxin detection and effector-triggered immunity can induce innate immune training. The following questions could further strengthen the study:

1. Universality of inflammasome-induced training

Is innate training a general consequence of inflammasome activation and IL-1 secretion? For example, can it be induced by other inflammasomes activated by bacterial signals (e.g., flagellin, dsDNA) or by sterile NLRP3 activators such as ATP? Is training enhanced by co-detection of alarmins released during lytic cell death?

We thank the reviewer for these question that help us to improve our manuscript. We had already included in Fig2 S-U that the trained phenotype was also observed by using Nigericin (a pore-forming toxin known as classical NLRP3 trigger). To extend our discovery to other inflammasomes we took advantage of a known trigger of the NLRC4 inflammasome namely the recombinant Needle protein (a protein of bacterial type III secretion systems) and observed that a first stimulation of the NLRC4 inflammasome induces trained immunity with an increased expression of Il-1b, IL-6 and Tnf-a. Therefore, triggering NLRC4 inflammasome is also able to induce trained immunity broadening the spectrum of our discovery. We added the panels V, W, X to figure 2, with the corresponding text (p6, line 155-161) as well as in the discussion (p11, line 304-310).

2. IL-1β versus IL-18 signalling

Recombinant IL-1β alone appears sufficient to induce monocyte training, whereas IL-18 does not, despite overlapping downstream signalling pathways. Do monocytes/macrophages in these cultures express IL-18 receptor?
To directly address the reviewer's question, we assessed IL-18R expression in BMDMs by Western blot analysis. We confirmed the presence of IL-18R protein in these cells, as shown in the blot below. In addition, Il-18r mRNA expression was detected by RT-qPCR, with the amplified products visualized by agarose gel electrophoresis. Together, these results confirm that our cultured BMDMs express IL-18R.

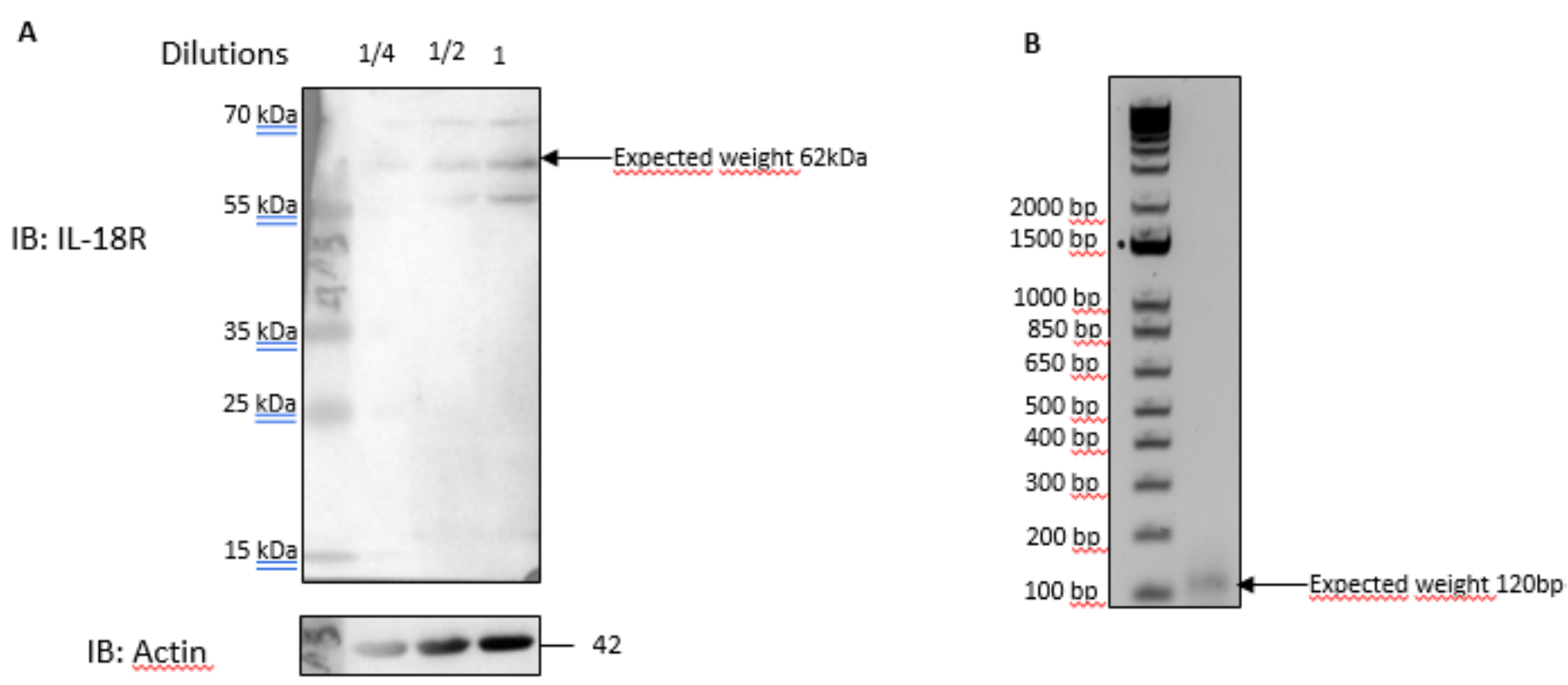

BMDM cells express IL-18 receptor

A. BMDM cells were lysed and processed for anti-IL-18R1 western blot. B. BMDMs were processed to analyse *IL-18R1* expression by RT-PCR.

3. Specificity of the training signature

ATAC-seq identifies genes induced by toxin training. Is this transcriptional/epigenetic signature shared with other training models (e.g., BCG or β-glucan), or is it stimulus-specific?

To address this question, we took advantage of public RNA seq data published by Christ A, *et al. Cell* 2018. This paper shows that western diet triggers NLRP3-dependant trained immunity. We found no correlation between western diet training and Bcl2a1 expression compared to control diet, suggesting that the signature we observed might be stimulus specific or at least not triggered by a sterile stimulus like western diet but further studies would be necessary to clearly demonstrate this point.

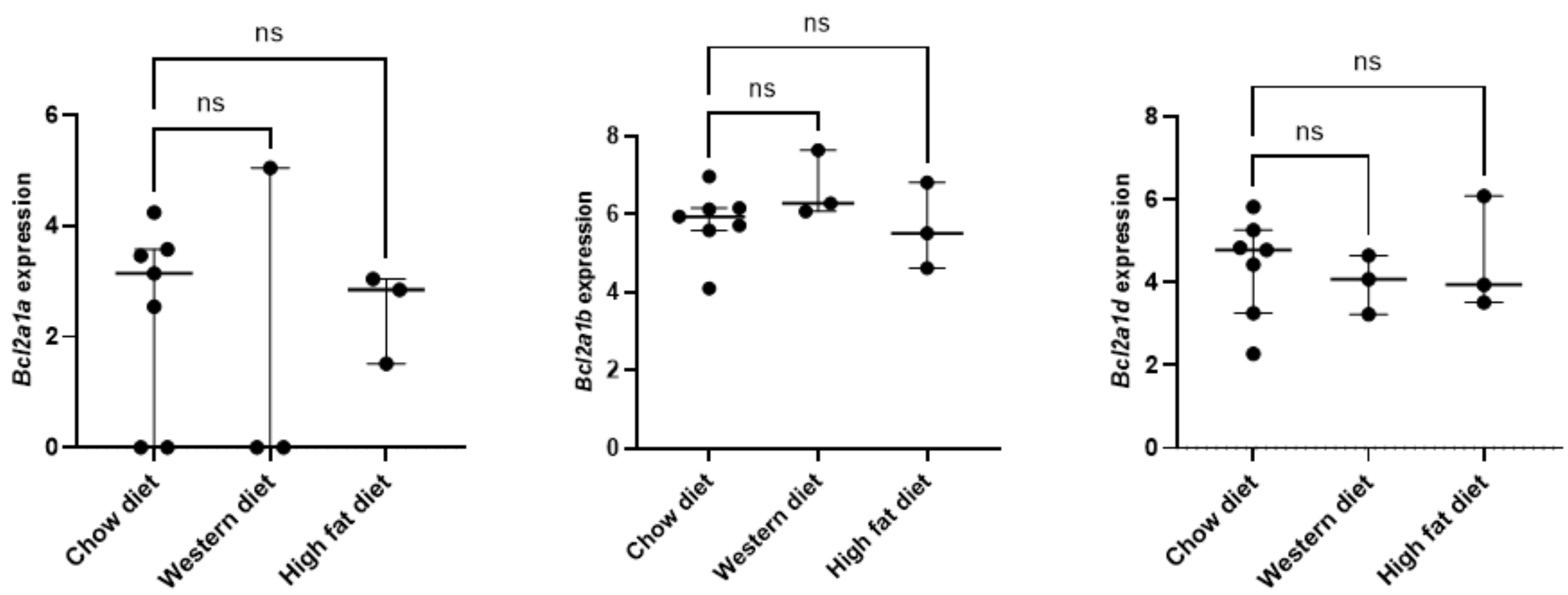

Analysis of RNAseq data to compare Bcl2a1 expression in mice fed with different diets
Expression levels of the Bcl2a1 gene family (Bcl2a1a, Bcl2a1b, Bcl2a1d) across experimental conditions Chow diet, Western diet, and Hight Fat diet all treated with LPS. Bcl2a1c was not detected. Expression values are shown on a log2-normalized scale. Boxplots represent median and interquartile range, with individual samples overlaid as points. Reanalysis from GSE106789 and GSE97926.

4. Role of Bcl2a1 in training
Toxin-triggered training is partially dependent on Bcl2a1. Is Bcl2a1 also required for BCG- or β-glucan–induced training, or is its function specific to toxin-mediated training?
We thank the reviewer for this important question. To directly address this point, we compared the trained immune response of wild-type and Bcl2a1-knockout BMDMs using our in vitro model of trained immunity. Unfortunately, under our experimental conditions, β-glucan did not induce a robust trained immunity phenotype. We only observed an increase in IL-1β secretion in wild-type macrophages at 6h after LPS induction, which was absent in Bcl2a1-deficient cells. In contrast, no increase in IL-6 or TNFα production was detected following β-glucan training. No changes were observed at 24h nor in ELISA or qPCR suggesting that our current experimental conditions are not optimal for capturing the kinetics of β-glucan-induced training and that extensive optimization would be required to define the appropriate induction and analysis time points. As these findings are only partial and do not allow us to draw firm conclusions regarding the role of Bcl2a1 in β-glucan-induced trained immunity, we decided not to include these data in the revised manuscript, as they fall outside the main scope of the present study.

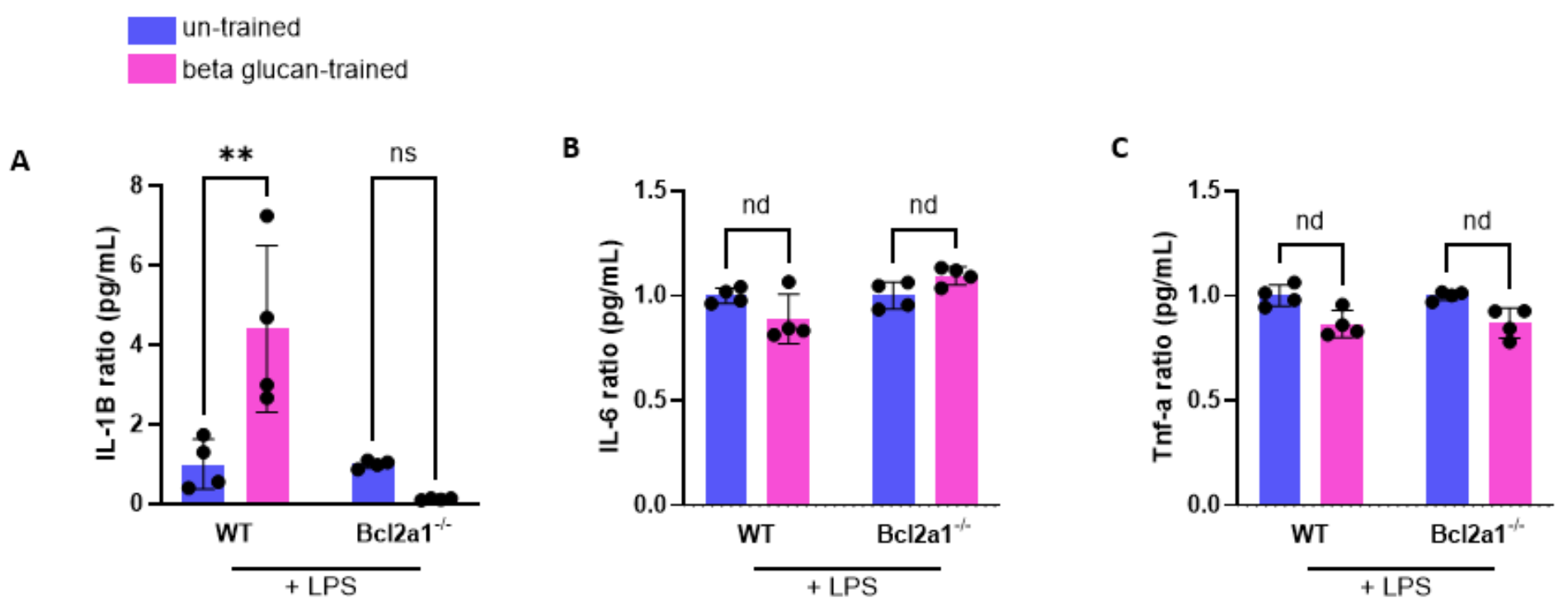

Bcl2a1 in beta-glucan-induced trained immunity

BMDM isolated from WT C57BL/6J or KO Bcl2a1 mice were trained with or without beta-glucan (1µg/mL) for 18 h at day 2. At day 8, cells were treated with LPS for 6h (A, B, C) (10 ng/mL). Levels of IL-1B (A), IL-6 (B) and Tnf-a (C) were assessed in the medium.

5. Interaction with NLRP3
The potential interaction between Bcl2a1 and NLRP3 is intriguing. Does Bcl2a1 interact with the NACHT domain of other NLR family members, or is this interaction unique to NLRP3?

We performed co-immunoprecipitations with Bcl2a1 and NLRP3, NLRP1 or NLRP10 inflammasomes presenting a NACHT domain. We also performed a co-immunoprecipitation with Bcl2a1 and Pyrin inflammasome which does not possess a NACHT domain. We were unable to detect any interaction between Bcl2a1 and these three other inflammasomes. This indicates that the interaction with Bcl2a1 might be specific to NLRP3.
These results have been added in the text (p8 225-229) and new supplementary Figure 3.

6. Duration and anatomical site of memory
How long does toxin-induced memory persist in vivo?

We thank the reviewer for the question. We have extended our analysis up to 4 weeks and now we showed that the cytokine responses induced by CNF1 training are transient and no longer statistically significant at 3 and 4 weeks post-training (Supplemental Figure 5). This point is now included in the manuscript (p9, 249-259)

Does training occur primarily in bone marrow progenitors or in tissue-resident macrophages, i.e. does memory persist after transplantation of trained bone marrow into naïve recipients?

To address this point, we have performed the requested experiment of bone marrow transfer (BMT) from trained mice into naïve recipients (see detailed protocol in figure legend below). Four weeks following bone marrow transfer (allowing bone marrow engraftment and spleen immune cells replenishment), spleen macrophages were extracted and stimulated *ex vivo*. Bone marrow transfer (BMT) efficiency was assessed by flow cytometry. BMT did not allowed to establish the maintenance of trained immunity in recipient mice, qPCR data following second stimulation *ex vivo* are represented in the figure below A, B and C.
We believe that the experimental setting of the BMT that requires 4 weeks to reconstitute the hematopoietic niche is not allowing us to prove this point since 4 weeks following CNF1 injection in donor mice was probably too late to maintain trained immunity.

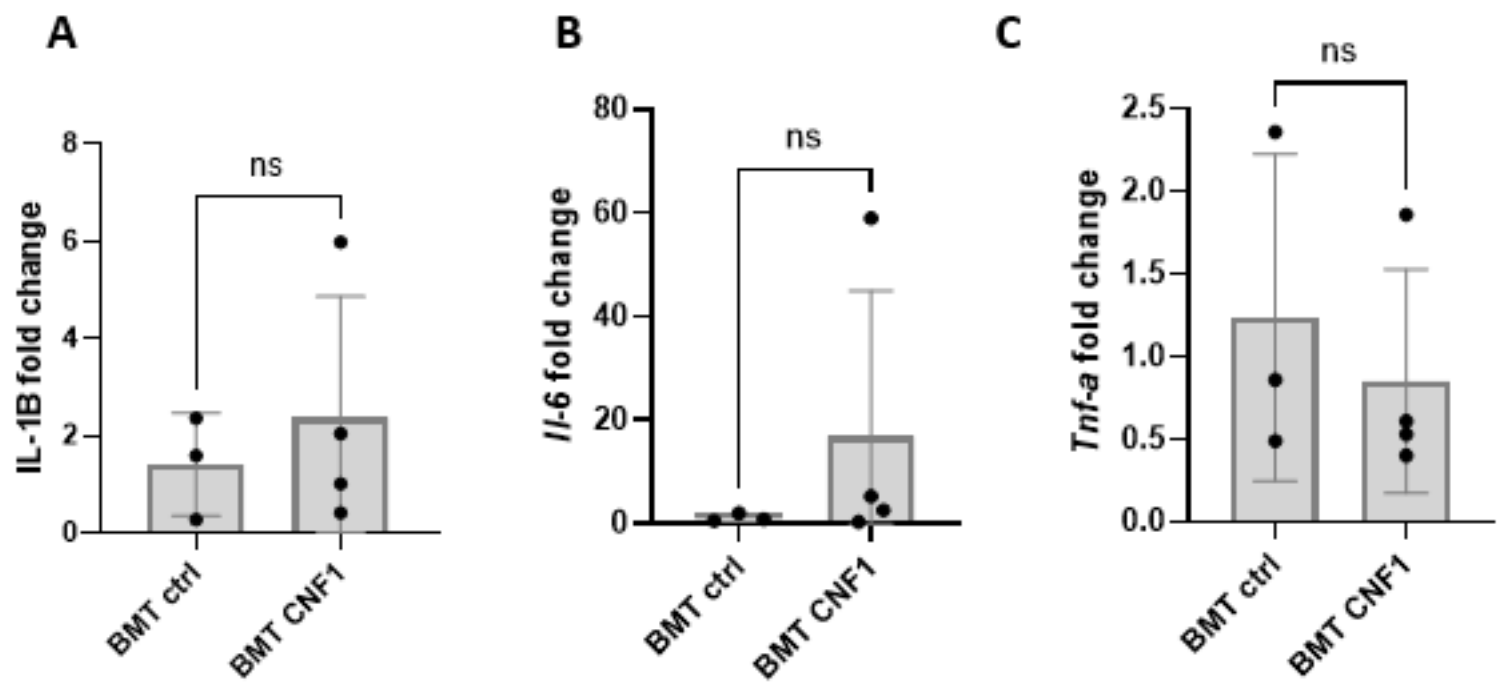

Clues on the anatomical site of innate immune memory

(A – C) ASC citrine C57BL/6J mice were trained with intravenous injection of WT CNF1 or its catalytic inactive mutant ($CNF1^{C866S}$) (250 ng/kg). A week after, bone marrow of these trained mice were engrafted in recipient irradiated C57BL/6J mice. Four weeks after, splenic macrophages were isolated, cultured for a week and then treated with LPS for 24 h (10 ng/mL). Gene expression were analyzed using qPCR for *Il1b* (A), *Il6* (B) and *Tnfa* (C), expressed in fold change. BMT: Bone Marrow Transfer

7. Timing of the training window
Is there a defined temporal window during which IL-1 signalling induces training in vivo after toxin detection? For example, in stroke models, IL-1 trains bone marrow progenitors only within the first week; blockade during that window prevents training, whereas later inhibition of IL-1 does not (PMID: 39043180)

We thank the reviewer for this interesting question. To define the temporal window during which IL-1β signaling contributes to the establishment of trained immunity, we used IL-1 receptor antagonist (IL-1RA) to block IL-1β signaling at different time points.

WT C57BL/6J mice were trained by intravenous injection of wild-type CNF1 and received IL-1RA either 1 hour before CNF1 administration, 3 days after CNF1 injection, or 9 days after CNF1 injection.

Although the variability between animals prevented us from obtaining sufficiently robust data to include these experiments in the revised manuscript, we provide these preliminary observations to address the reviewer's question. Administration of IL-1RA 1 hour before CNF1 injection markedly reduced Il1b, Il6, and Tnfα expression following secondary challenge, reaching statistical significance only for Il6. In contrast, administration of IL-1RA at 3 or 9 days after CNF1 injection had no detectable effect, as cytokine expression remained comparable to that observed in CNF1-trained mice.

These results suggest that early IL-1β signaling might be necessary for the establishment of CNF1-induced trained immunity. This finding is consistent with the work of Alba Simats et al. *Cell* (2024), that showed in a mouse model of stroke that blockade of IL-1β during the acute phase prevented the increase in myelopoiesis responsible for subsequent cardiac pathology. A more precise characterization of this temporal window will require additional experiments and large animal cohorts, which could not reasonably be completed within the timeframe allotted for the revision.

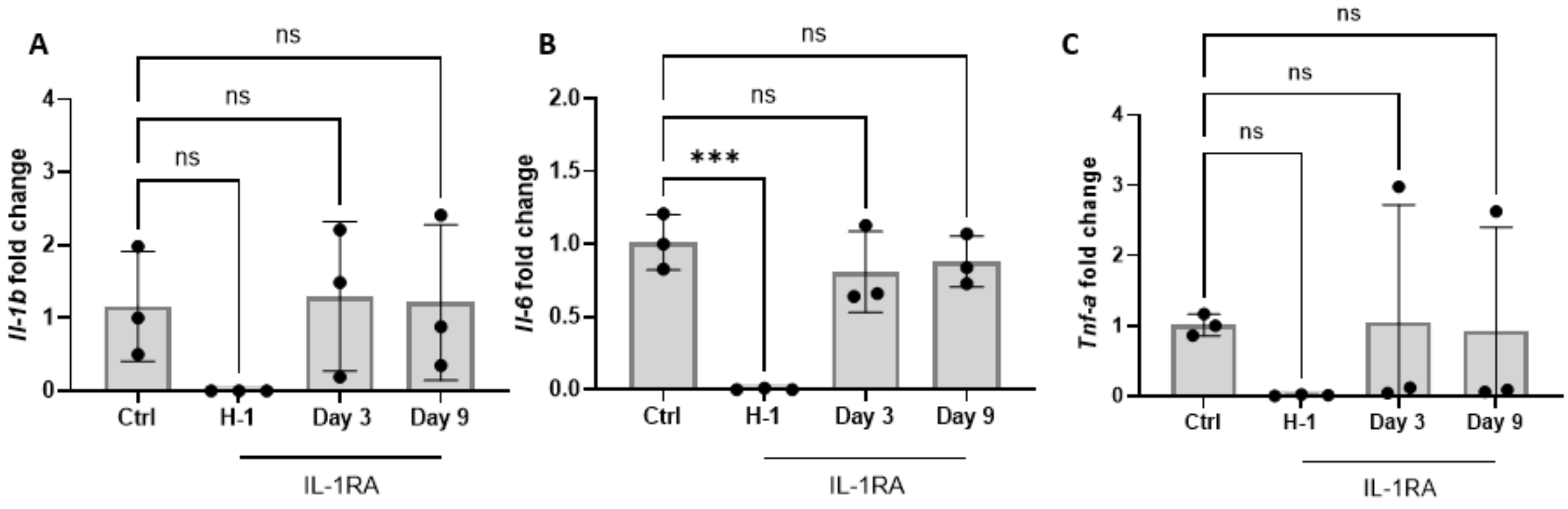

Importance of IL-1B signalling just after the first training stimulation

(A – C) WT C57BL/6J mice were trained with intravenous injection of WT CNF1 (250 ng/kg). These mice were also injected with IL-1RA one hour before CNF1 injection, or 3 days after CNF1 injection, or nine days after CNF1 injection. Two weeks after, splenic macrophages were isolated, cultured for a week and then treated with LPS for 24 h (10 ng/mL). Gene expression were analyzed using qPCR for *Il1b* (A), *Il6* (B) and *Tnfa* (C), expressed in fold change.

8. Risk of immunopathology

Could toxin-induced training predispose developing monocytes to exaggerated responses to non-infectious triggers (e.g., MSU crystals in vivo)? If so, this might increase susceptibility to inflammatory comorbidities, similar to what was observed after stroke-induced innate training. Are there any data on this in the human population that could be analysed (I understand this may not be available)?

We thank the reviewer for raising this important point. We agree that toxin-induced trained immunity may not only enhance host protection against subsequent infections but could also increase susceptibility to excessive inflammatory responses to sterile triggers. Although we did not directly test whether CNF1-induced training enhances responses to non-infectious stimuli such as MSU crystals, we agree that this possibility deserves careful consideration. Several studies have highlighted the long-term consequences of severe inflammatory insults and the potential contribution of maladaptive innate immune memory to chronic comorbidities. In particular, sepsis survivors exhibit increased risks of cardiovascular, renal, metabolic, and neurological diseases, suggesting that acute inflammatory episodes can have persistent effects on systemic homeostasis. A recent prospective cohort study describing disease trajectories after sepsis demonstrated that survivors followed distinct trajectories characterized by increased mortality and a higher incidence of chronic comorbidities affecting multiple organ systems, emphasizing that the consequences of acute inflammation extend far beyond the initial infectious episode. These observations are consistent with the concept that persistent innate immune reprogramming may contribute to long-term pathology. Similarly, Alba Simats et al. *Cell* (2024) showed that stroke-induced IL-1β-dependent trained immunity promotes inflammatory cardiac dysfunction, illustrating how acute inflammatory insults can reprogram myeloid cells and drive remote organ pathology. We have now incorporated this point into the Discussion. We propose that CNF1-induced trained immunity may represent a double-edged sword: beneficial in the context of subsequent bacterial infection, but potentially detrimental if trained myeloid cells encounter sterile inflammatory stimuli. At present, we do not have access to a sufficiently powered human cohort allowing us to determine whether infection with CNF1-producing *E. coli* is associated with an increased incidence of inflammatory comorbidities. Future clinical studies will be required to address this question (p11 line, 330-337).

Please find below our point-by-point responses to the reviewer's comments. We sincerely thank the reviewer for their thoughtful and constructive feedback, which has significantly improved our manuscript.

REVIEWER COMMENTS

Reviewer #1 (Remarks to the Author):

All previous points have been adequately responded to. No more comments.

Reviewer #2 (Remarks to the Author):

The authors have addressed all comments except one and significantly improved the manuscript.

The only comment that was not addressed was in the Overall section and asked for "Data showing the importance of Bcl2A1 for NLRP3 inflammasome activation". Unless I am missing something I could not find this experimental result in the revised manuscript. Unless the authors can argue otherwise I think this is an important experiment and straightforward since Bcl2a1-/- macrophages are available.

We apologize for not recognizing this statement as a specific request in our initial revision. To fully address the reviewer's comment, we performed the requested experiment using LPS + CNF1 to activate the NLRP3 inflammasome. We found that LPS + CNF1-treated Bcl2a1-/- macrophages secreted significantly higher levels of IL-1β than wild-type macrophages, whereas IL-6 secretion remained unchanged. These findings suggest that BCL2A1 may act as a negative regulator of NLRP3 inflammasome activation. We have included these new data in Supplementary Figure 2C–D and have revised the manuscript accordingly (lines 225–229, page 8).

Reviewer #3 (Remarks to the Author):

I thank the authors for their thorough and thoughtful revision. The revised manuscript is substantially improved and addresses the vast majority of the concerns raised during the first round of review.

I have one remaining conceptual concern regarding the interpretation of the findings that I believe should be addressed through important revisions to the text before publication.

Major comment

The revised manuscript convincingly demonstrates induction of an innate trained phenotype following CNF1 exposure. The combination of a resting phase between stimuli, enhanced secondary responses, epigenetic remodelling (ATAC-seq), transcriptional reprogramming, and IL-1/NLRP3 dependence is consistent with current definitions of trained immunity.

However, I do not believe the current data support conclusions implying durable central trained immunity or long-lived bone marrow-resident immune memory, as described for classical models such as BCG, β-glucan or Western diet-induced training.

The additional experiments introduced during revision show that:

1. the trained phenotype is detectable at 1–2 weeks but is no longer statistically significant at 3–4 weeks;
2. alterations in myeloid cell composition are similarly transient;
3. bone marrow transplantation experiments, which require 4 weeks to engraft, were unable to transfer the phenotype to naïve recipients.

Taken together, these observations suggest that CNF1 induces a transient trained immune programme rather than durable hematopoietic reprogramming comparable to that described following BCG or β-glucan exposure.

While bone marrow reprogramming is not a formal requirement for defining trained immunity, it has become a hallmark of persistent central in vivo trained immunity. The current study therefore does not establish that the long-term memory resides within long-lived hematopoietic progenitors.

Accordingly, I recommend that the authors moderate several statements throughout the manuscript.

We thank the reviewer for this insightful comment. We have addressed this point by revising the manuscript accordingly.

Specific suggestions:

1. The current title refers to "innate immune memory," which is acceptable. However, I recommend avoiding wording that may imply durable or long-lived memory. An alternative such as "Bacterial toxin sensing induces transient inflammasome-dependent trained immunity that improves survival during sepsis" may better reflect the data.

We fully agree with the reviewer and have modified the title as suggested.

2. I recommend modifying the final sentence of the Abstract to acknowledge the transient nature of the response. For example: These findings identify toxin-driven inflammasome activation as a trigger of transient trained immunity and demonstrate that this response enhances resistance during experimental bacterial sepsis.

We agree and have modified the abstract as suggested.

3. The Results section entitled "CNF1 toxin induces central TI and protects against lethal infection" appears stronger than warranted. A title such as "CNF1 induces transient myeloid reprogramming and protects against lethal infection" would be more consistent with the data. Likewise, the paragraph discussing the increase in CMPs should be softened. Statements suggesting that long-lived hematopoietic stem cells explain the persistence of immune memory are speculative and are not supported by the presented data, particularly in light of the transient phenotype and the inability of bone marrow transplantation to transfer the phenotype.

We agree with the reviewer. We have changed the title of the section and have softened the text as suggested.

4. The Discussion would benefit from explicitly acknowledging that the trained phenotype is reversible. Rather than presenting this as a limitation, I think this could become one of the conceptual strengths of the study. The authors could discuss the possibility that bacterial toxins induce a transient, self-limited form of trained immunity that provides short-term protection against recurrent infection while minimising the risks associated with prolonged inflammatory reprogramming. This interpretation would also fit well with the newly added discussion regarding potential immunopathology.

We thank the reviewer for this important comment. We have addressed this point by revising the Discussion accordingly.
